# Autophagy-Mediated Clearance of Free Genomic DNA in the Cytoplasm Protects the Growth and Survival of Cancer Cells

**DOI:** 10.3389/fonc.2021.667920

**Published:** 2021-05-26

**Authors:** Mengfei Yao, Yaqian Wu, Yanan Cao, Haijing Liu, Ningning Ma, Yijie Chai, Shuang Zhang, Hong Zhang, Lin Nong, Li Liang, Bo Zhang

**Affiliations:** ^1^ Department of Pathology, School of Basic Medical Sciences, Peking University Health Science Center, Beijing, China; ^2^ Department of Pathology, Peking University First Hospital, Beijing, China

**Keywords:** autophagy, cGAS, Micronuclei, cytoplasmic DNA, breast cancer

## Abstract

The cGAS (GMP-AMP synthase)-mediated senescence-associated secretory phenotype (SASP) and DNA-induced autophagy (DNA autophagy) have been extensively investigated in recent years. However, cGAS-mediated autophagy has not been elucidated in cancer cells. The described investigation revealed that active DNA autophagy but not SASP activity could be detected in the BT-549 breast cancer cell line with high micronucleus (MN) formation. DNA autophagy was identified as selective autophagy of free genomic DNA in the cytoplasm but not nucleophagy. The process of DNA autophagy in the cytosol could be initiate by cGAS and usually cooperates with SQSTM1-mediated autophagy of ubiquitinated histones. Cytoplasmic DNA, together with nuclear proteins such as histones, could be derived from DNA replication-induced nuclear damage and MN collapse. The inhibition of autophagy through chemical inhibitors as well as the genomic silencing of cGAS or SQSTM1 could suppress the growth and survival of cancer cells, and induced DNA damage could increase the sensitivity to these inhibitors. Furthermore, expanded observations of several other kinds of human cancer cells indicated that high relative DNA autophagy or enhancement of DNA damage could also increase or sensitize these cells to inhibition of DNA autophagy.

## Introduction

cGAS is an enzyme that catalyzes GTP and ATP to form cyclic dinucleic 2′,3′-cGAMP to stimulate stimulator of interferon gene (STING) and activate the kinases TBK1 and IKK, inducing the production of proinflammatory factor type I interferon (1). cGAS-STING has been identified as an innate immune mechanism, but many recent studies have shown that cGAS-STING plays a major role in the activation of the senescence-associated secretory phenotype (SASP), in which senescent cells secrete many cytokines, growth factors, proteases and chemokines acting through either autocrine or paracrine mechanisms to promote inflammation (2–6). The SASP is a crucial biological factor involved in aging-related diseases such as chronic inflammation, tissue degeneration or organ retardation. More importantly, the SASP has been revealed to be responsible for preventing the growth of cancer cells through its role as a tumor suppressor in the late stages of tumor progression *via* genomic instability of cancer cells and remodeling of the tumor microenvironment (7). More interestingly, recent studies have also demonstrated that cGAS-STING could induce autophagy upon binding to dsDNA through either the cGAS interaction with Beclin-1 or STING-mediated LC3 lipidation. Nevertheless, either SASP (proinflammatory) or autophagy is important in host innate defense (8). However, cGAS-STING-mediated SASP or autophagy have not been fully elucidated in cancer cells.

Activation of cGAS has been confirmed upon its binding to DNA, and *in vitro* analysis proved that DNA could effectively recruit cGAS through phase transition (9). In living cells, cytoplasmic DNA, which can be exogenous, such as that from pathogenic organisms, and endogenous DNA of host cells, is a trigger that activates cGAS. Micronuclei (MNs) are small nuclei separated from the main nucleus. Similar to the main nucleus, MNs are encapsulated by the nuclear membranes and contain DNA and related substances (10). MNs are prevalent in cancer cells and are believed to be a consequence of DNA damage and aberrations in mitosis (11, 12). Moreover, studies have suggested that the existence of cytosolic DNA is closely related to MNs (6). MNs have been identified as the major source of cytoplasmic DNA involved in the activation of the cGAS-STING machinery to promote cancer progression and metastasis (13–15). Moreover, free DNA derived from ecc rDNA (extrachromosomal circular rDNA) could also trigger cGAS-STING activity (16). In addition, endogenous cytoplasmic DNA could be derived from mitochondria, which are injured under many circumstances (17). Nevertheless, in contrast to its role in triggering the SASP phenotype by free DNA, cGAS-STING-mediated DNA autophagy in cancer cells has rarely been evaluated.

The mechanism by which the cGAS-STING pathway mediates either SASP or autophagy in response to exogenous pathogens could lead to their eradication. However, it is unclear how cGAS-STING makes decisions in response to endogenous DNA in cells. In the described investigation, we unexpectedly found that the BT-549 breast cancer cell line with a high frequency of MN formation presented a low SASP phenotype but high autophagic activity, and subsequent experiments showed that its high DNA autophagy mediated by cGAS and cytosol-free DNA was closely related to MN formation and DNA damage, and inhibition of DNA autophagy could suppress its growth and survival. Furthermore, expanded observations indicated that enhancement of DNA damage or cancer cells with high relative DNA autophagy could increase DNA autophagy and sensitize the cells to autophagic inhibitors. These results proved that SASP and autophagy were related to the extent of DNA damage and that severe DNA damage or deficient DNA damage repair could increase autophagy in cells. Our research also clarified the potential therapeutic role of autophagic inhibition in some kinds of cancer cells with extensive DNA damage.

## Materials and Methods

### Reagents and Antibodies

The anti-Lamin B1 rabbit polyclonal antibody, anti-Beclin1 rabbit polyclonal antibody, anti-STING (TMEM173, EPR13130) rabbit monoclonal antibody, and anti-DNase2 rabbit monoclonal antibody were purchased from Abcam (Cambridge, UK). The Stat6 (D-1) mouse monoclonal antibody, Lamin B1 mouse monoclonal antibody and cGAS (D-9) were purchased from Santa Cruz Biotechnology, Inc. (CA, 95060, USA), and anti-phospho-histone γH2AX mouse monoclonal antibody (Ser139) and anti-RPA2 mouse monoclonal antibody were from Millipore (Billerica, MA, USA). The anti-Lamin A/C (R386) rabbit polyclonal antibody, anti-IRF3 rabbit polyclonal antibody and anti-LAMP2 rabbit polyclonal antibody were from Bioworld Technology, Inc. (MN, USA). Anti-SQSTM1 rabbit polyclonal antibody and anti-LC3 rabbit polyclonal antibody were purchased from MBL, Ltd. (Chiba, Japan). Anti-phospho-STING (Ser366) rabbit monoclonal antibody and phospho-IRF3 rabbit monoclonal antibody were purchased from Cell Signaling Technology (Danvers, MA, USA). The information about antibodies were summarized in **Supplementary Table 1**. Nuclear Fast Red Staining Solution (0.1%; G1320), LysoTracker staining kit (Lyso-tracker Red DND-99) (L8010) and DAPI (C0060) were purchased from Solarbio (Beijing, China). The full-length expression plasmids Flag-cGAS, Flag-SQSTM1 and Flag-Beclin-1 were purchased from YouBio, Inc. (Beijing, China). Bafilomycin A1 and H-151 were purchased from Selleck (Shanghai, China), and chloroquine (CQ) and 2,3’-cGAMP were purchased from Sigma Aldrich (St. Louis, MO, USA). The protein marker (PM2510) was purchased from SMOBIO (Taiwan).

### Cell Culture and Treatment

MDA-231, MCF-7, BT-549, 786-0, DU145, PC-3M, HCT-116, and HeLa cancer cell lines were maintained in 1640 or Dulbecco’s modified Eagle’s medium with high glucose (Gibco, Life Technologies, Grand Island, NY, USA) supplemented with 10% fetal bovine serum. The cells were incubated in a humidified atmosphere with 5% CO_2_ at 37°C.

### Small Interfering RNA

RNAi was designed and synthesized by GenePharma (Suzhou, China). RNAi was performed by the transfection of siRNA oligos using Lipofectamine 2000 transfection reagent (Invitrogen) according to the manufacturer’s instructions. The sequences are as follows: si-cGAS-1: Forward: 5’-GGCCUCUGCUUUGAUAACUTT-3’, Reverse: 5’-AGUUAUCAAAGCAGAGGCCTT-3’; si-cGAS-2: Forward: 5’-GGCUAUCCUUCUCUCACAUTT-3’; Reverse: 5’ –AUGUGAGAGAAG GAUAGCCTT-3’. si-LC3-1: Forward: 5’-GCUACAAGGGUGAGAAGCATT-3’; Reverse: 5’-UGCUUCUCACCCUUGUAGCTT-3’; si-LC3-2: Forward: 5’-GCGAGUUGGUCAAGAUCAU TT-3’; Reverse: 5’-AUGAUCUUGACCAACUCGCTT-3’; si-LC3-3: Forward: 5’ –GCUUCCUC UAUAUGGUCUATT-3’; Reverse: 5’-UAGACCAUAUAGAGGAAGCTT-3’. si-SQSTM1-1: Forward: 5’-AGAUUCGCCGCUUCAGCUUTT-3’; Reverse: 5’-AAGCUGAAGCGGCGAAUC UTT-3’; si-SQSTM1-2: Forward: 5’-CGCUCACCGUGAAGGCCUATT -3’; Reverse: 5’-UAGGC CUUCACGGUGAGCGTT-3’; si-SQSTM1-3: Forward: 5’-GCACUACCGCGAU GAGGACTT-3’; Reverse: 5’-GUCCUCAUCGCGGUAGUGCTT-3’; si-DNase II-1: Forward: 5’-GGCAGCCU GUAGACUGGUUTT-3’; Reverse: 5’ -AACCAGUCUACAGGCUGCCTT-3’; si-DNase II-2: Forward: 5’-GCAUACAGCUGGCCUCAUATT-3’; Reverse: 5’-UAUGAGGCCAGCUGUAUG CTT-3’. The control RNAi (siNC) was composed of scrambled sequences.

### Western Blotting

Total cell lysates were obtained by incubating the cells in 2× SDS for 30 minutes at 4°C. After centrifugation at 10,000×g for 10 minutes at 4°C, the supernatant was collected and stored at -20°C for subsequent analysis. For cell fractions, cytoplasmic and nuclear proteins were extracted using nuclear and cytoplasmic protein extraction kits (Sangon Biotech Co., Shanghai, China), respectively. Equal amounts of cell proteins (20-40 μg/lane) were separated by SDS-PAGE in 10% gels and transferred to PVDF membranes (Millipore, Billerica, MA, USA) using a semidry transfer cell (Bio-Rad,Hercules, CA, USA) at 25 V for 60 minutes. The membranes were then blocked for 1 hour with TBS-T (20 mmol/L Tris-HCl pH 7.6, 137 mmol/L NaCl and 0.1% Tween-20) containing 5% nonfat dry milk (Cell Signaling Technology, Beverly, MA, USA) or with 1% BSA (Sigma Aldrich) and incubated overnight with primary antibodies. After the membranes were washed, they were incubated for 1 hour with peroxidase-conjugated goat anti-rabbit IgG or peroxidase-conjugated goat anti-mouse IgG. The proteins were visualized using an enhanced chemiluminescence kit (Bio-Rad, CA, USA). Band images of three independent experiments were quantified by optical density using Lab-Works 4.6 software (Bio-Rad, CA, USA). β-actin was used as an internal control for each protein. The antibodies included anti-LC3 (1:1000), anti-β-actin (1:1000), anti-cGAS (D-9) (1:500), anti-SQSTM1 (1:1000), anti-DNase2 (1:1000), anti-lamin A/C (R386) (1:500), anti-IRF3 (1:500), anti-TMEM173 (1:1000), and anti-LAMP2 (1:1000).

### Immunofluorescence

Immunofluorescence staining was performed as described previously (18). The results were observed and recorded using a fluorescence microscope (Model CX51; Olympus, Tokyo, Japan), and Photoshop version 7.0 (Adobe Systems, Inc.) was used to analyze the results. The antibodies used included anti-LC3 (1:1000), anti-β-actin (1:1000), anti-cGAS (D-9) (1:500), anti-SQSTM1 (1:1000), anti-DNase II (1:1000), anti-lamin A/C (R386) (1:500), IRF3 (1:500), anti-STING (1:1000), and anti-LAMP2 (1:1000).

### Senescence Associated-β-galactosidase Staining (SA-β-gal)

Cells were subjected to SA-β-gal staining using an SA-β-gal staining kit (GenMed, GenMed Scientifics, Inc., USA) according to the manufacturer’s directions. Five hundred cells were counted in random fields and the percentage of SA-β-gal-positive cells was calculated and the mean was calculated from independently repeated experiments at least five times.

### Electron Microscopy

Cells were fixed with 0.1 mol/L sodium phosphate buffer (4% paraformaldehyde and 0.1% glutaraldehyde) at 4°C overnight. The samples were embedded with Lowicryl K4M (BioChemika), and sections were prepared with the Leica EM UC7 ultramicrotome and then stained with saturated uranyl acetate. The sections were observed under a transmission electron microscope (JEOL-1230, Japan) and recorded. The autophagosomes or autolysosomes are double-membrane enclosed in EM observations with the treatment of autophagy inhibitors. 

### Co-Immunoprecipitation

A total of 1x10^7^ transfected cells were washed twice with PBS, 600 μl of precooled hypo-osmic buffer (25 mmol/L Tris, pH 7.4, 85 mmol/L KCl) was added, and the samples were incubated on ice for 30 minutes and centrifuged at 4°C at 12000 rpm for 10 minutes. The supernatant was saved and incubated with Flag antibody-conjugated agarose beads (MBL, Chiba, Japan) and gently shaken on a turntable overnight at 4°C. The beads were washed with hypo-osmic buffer containing protease inhibitor cocktail for 10 minutes; this process was repeated 4 times. Finally, the beads were dissolved in 1.5×SDS loading buffer, and 30 μl of supernatant was analyzed by Western blotting. The primary whole cytoplasmic supernatant was used as input.

### IP-PCR

A total of 2×10^7^ transfected cells were washed with preheated PBS at 37°C 3 times, fixed with 1% formaldehyde in PBS in a 37°C incubator for 15 minutes, quickly washed with ice-cold PBS 5 times, scraped into an Ep tube, and centrifuged at 800 g at 4°C for 3 minutes. Then, the supernatant was discarded, and 500 μl of hypo-osmic buffer (25 mmol/L Tris, pH 7.4, 85 mmol/L KCl) was added to isolate the cytoplasmic protein. Part of the supernatant was saved as the input. The remaining part was used for IP experiments. An appropriate amount of 500 μl elution buffer (1% SDS, 0.1 mol/L sodium bicarbonate was used to elute the protein-DNA complex on the beads for 10 minutes at room temperature. Then, RNase A was added, the samples were heated at 37°C and shaken for 2 h, Proteinase K was added, and the samples were heated at 55°C and shaken for 2~3 h. Then, the samples were heated at 65°C and shaken overnight to isolate the protein-DNA complexes. Finally, the IP DNA was extracted by the phenol and chloroform method, and the results were analyzed by PCR.

### Sucrose Density Gradient Centrifugation

Gradient concentrations of sucrose solution (5%-40%, the concentration interval was 5%) with protease inhibitor cocktail were established as described (19). The cytoplasmic proteins, extracted by hypo-osmic buffer as described above, were carefully dropped on the top layer and centrifuged at 40,000 rpm (Beckman, Brea, CA, USA) for 4 hours at 4°C. After centrifugation, the samples were carefully collected from a 500 μl aliquot of each fraction, and the aliquot of each fraction was analyzed by Western blot. Cytoplasmic DNA was extracted from 100 μl of each fraction and analyzed by PCR.

### Acid Extraction of Cytoplasmic Histones

Cytoplasmic histones were isolated by acid extraction methods with some modifications (20). Briefly, cytoplasmic proteins from 1×10^7^ cells of each kind, extracted by hypo-osmic buffer as described above, were slowly added to 0.4 N H_2_SO_4_ (500 μl of H_2_SO_4_ to a 100 μl cytoplasmic solution) and incubated at 4°C with intermittent rotation for 2 hours. After centrifugation at 5500 rpm for 5 minutes, the supernatants were gently added to 150 μl of 100% TCA (final concentration of 20%) and kept on ice for at least 5 hours without agitation. After centrifugation, the pellets were washed with 500 μl of acetone+0.1% HCl, and the tubes were left open overnight to evaporate the acetone. The pelleted histones were resuspended in 20 μl of ddH_2_O and subsequently analyzed by SDS-PAGE and Western blotting.

### PCR Detection

DNA in nuclear or cytoplasmic were separately extracted. The cells were cultured in 10 cm dishes and counted. A total of 1×10^3^ cells were treated with hypo-osmic buffer (25 mmol/L Tris pH 7.4, 85 mmol/L KCl) and centrifuged, and the supernatant (the cytoplasmic components) was saved. Then, the cytoplasmic DNA was extracted by the phenol and chloroform method. The sediment (nuclear DNA) was extracted by Tigen Kits (Beijing, China). PCR was performed with using Alu and rDNA primers: Alu: sense: 5’-CCTGAGGTCAGGAGTTCGAG-3’; antisense: 5’-CCCGAGTAGCTGGGATTACA-3’ (115 bp); 5.8S rDNA: sense: 5’- GAGGCAACCCCCTC TCCTCTT-3’; antisense: 5’-GAGCCGAGTTCACCGCTA-3’ (136 bp); 18S rDNA: sense: 5’-CG CCGCGCTCTACCTTACCTA-3’; antisense: 5’ -TAGGAGAGGAGCGAGCGACCA-3’ (159 bp); 28S rDNA: sense: 5’ -CTCCGAGACGCGACCTCAGAT-3’; antisense: 5’-CGGGTCTTCC GTACGCCACAT-3’ (173 bp). Quantitative real-time RT-PCR was performed on a PCR system (Applied Biosystems, Inc., Carlsbad, CA, USA) using SYBR Premix. The results were evaluated as the ratio of cytoplasmic: nuclear DNA; cytoplasmic DNA was normalized to nuclear DNA. The results were analyzed by GraphPad Prism 8.0.

### FISH

The Alu probes were as follows: Alu-1: 5’-CCTGAGGTCAGGAGTTCGAGACCAGCCT-3’; Alu-2: 5’-ACGCCTGTAATCCCAGCACTTTGGGAGG-3’; Alu-3: 5’ –TCGCGCCACTGCACTC CAGCCTGGGCGA-3’. They were synthesized and conjugated with a single Quasar 570 molecule at the 5’ end (Sango Biotech, Shanghai, China). Cells grown on cover slides were fixed in 3% paraformaldehyde (pH 7.4) containing 0.1% Triton-X 100 for 30 minutes and permeabilized in 0.1% Triton X-100 for 20 minutes. After denaturation at 95°C for 5 minutes, hybridization was performed in a mixture of probes (20 ng/per slide) and 35% deionized formamide, 10% dextran sulfate, 1× Dehart’s, and 2× SSC for 20 hours at 42°C. The slides were washed for 40 minutes in 2×SSC with several changes. Nuclei were stained with Hoechst 34580 (Sigma Aldrich) for 10 minutes, and the results were observed and recorded using a fluorescence microscope (Model CX51, Olympus, Tokyo, Japan), and Photoshop version 7.0 (Adobe Systems, Inc. San Jose, CA, USA) was used to observe and analyze the results. At least 500 cells were evaluated, and the results were evaluated as the ratio of the intensity in cytoplasmic and nuclear DNA; cytoplasmic signals were normalized to those of the nuclei.

### Comet Assay

The comet assay was performed using a comet assay kit according to the manufacturer’s instructions. First, 1×10^5^ cells were prepared. Neutral or alkaline electrophoresis was performed. The slides were viewed by epifluorescence microscopy using a FITC filter. The results were analyzed by Comet Score software (Version 2.0038).

The comet assay was performed using a comet assay kit (Abcam, Cambridge, UK) according to the manufacturer’s instructions. First, 1×10^5^ cells were prepared. Comet Agarose was pipetted onto the Comet Slide to form base layer cells, which were combined with Comet Agarose at 37°C. Cell samples were combined with Comet Agarose at a 1/10 ratio (v/v). We pipetted the agarose/cell mixture on top of the base layer. The cells were treated with lysis buffer and alkaline solution. Electrophoresis was performed under alkaline or neutral conditions. Voltage was applied to the chamber for 10-15 minutes at 1 volt/cm. The cells were stained with DNA dye. The slides were viewed by epifluorescence microscopy using a FITC filter. The results were analyzed by Comet Score software (Version 2.0038).

### BrdU Incorporation Assay

BrdU incorporation assays were performed as described previously (18). Briefly, BrdU (10 µM) was added to the culture medium for 30 minutes before analysis, and then, the cells were fixed with 4% formaldehyde, permeabilized with 0.1% Triton X-100, and denatured with 20 mmol/L HCl in 150 mmol/L NaCl and 3 mmol/L KCl for 20 minutes at 25°C. The cells were then incubated with a primary antibody mixture composed of primary antibodies (BrdU and MCM7 or Lamin B1).

### Trypan Blue Exclusion Assay

Cell growth was determined by Trypan blue exclusion assays with a Trypan Blue Staining Cell Viability Assay Kit (Beyotime, Shanghai, China). Cells (1 ×10^4^ cells/per well) grown with or without treatment with CQ, bafilomycin A1, or H-151 in 96-well plates were harvested, and 50 μl of trypan blue was added to a 50 μl cell suspension according to the manufacturer’s protocol. Viable cells were counted under a microscope with a hemocytometer. The assays were performed in triplicate and repeated at least three times.

### Live/Dead Viability Assay

A live/dead assay was performed using a live/dead cell viability assay kit (Abcam, Cambridge, UK). A total of 1×10^5^ cells were seeded in 12-well or 96-well plates and incubated for 24 hours. The cells were treated with CQ or bafilomycin A1 and incubated for the indicated times. Subsequently, the cells were rinsed twice with PBS before the fluorochromes were added and incubated for 45 minutes. Fluorescence images were then taken (Model CX51, Olympus, Tokyo, Japan), and live or dead cells were counted and calculated.

### MTS Assay

MTS assays were performed using an MTS assay kit (Bestbio, Shanghai, China). A total of 1×10^4^ cells were seeded in 96-well plates and incubated for 24 hours. The cells were treated with CQ or bafilomycin A1 and incubated for the indicated times. Ten microliters of MTS solution was added to each well and incubated for 3 hours at 37°C. The absorbance was measured at 490 nm, and cell viability was analyzed.

### Statistical Analysis

All analyses were performed using software GraphPad Prism 8 (CA, USA), SPSS 27.0 (IBM, Armonk) and Excel 2010 (WA, USA). Comparisons between groups were performed using Student’s t-test and one-way ANOVA with Tukey *post-hoc* test and Dunnett *post-hoc test* to correct for multiple testing. The data were presented as mean ± SEM or mean ± SD. A P-value of less than 0.05 was considered significant. All statistical tests and P values were 2-sided, and the level of significance was set at <0.05 (*), <0.01 (**), <0.001 (***), or<0.0001 (****); ns indicates no significance.

## Results

### Detection of the Autophagy Activity in Breast Cancer Cells

**Figure 1:**
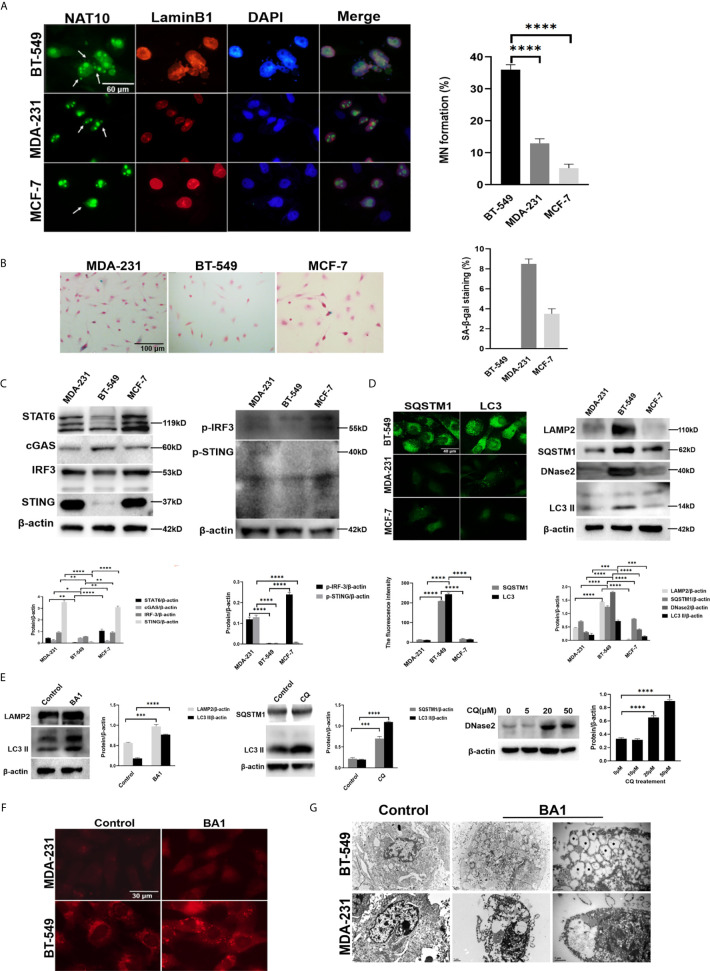
High frequency of MNs in breast cancer cells was associated with an autophagic phenotype. **(A)** MN formation in breast cancer cells. Left panels: MDA-231, BT-549 and MCF-7 cells were subjected to immunofluorescence staining for NAT10 and Lamin B1. Arrows indicate MNs. Right panel: the frequency of MNs in each kind of cell was counted and calculated as the percentage of total cells. The micronuclei outlined by LaminB1 and DAPI were counted and at each time 500 cells were measured. The experiments were repeated five times. The frequency of MN was calculated from independently repeated experiments. Data are presented as mean ± SEM. **(B)** Senescent phenotypes of breast cancer cells. Left panels: Representative images of SA-β-gal staining in the MDA-231, BT549 and MCF7 cells. Right panel: SA-β-gal-positive cells were counted and calculated as the percentage of total cells. The experiments were repeated five times. Data are presented as mean ± SEM. **(C)** The expression of SASP-related factors in breast cancer cells. The MDA-231, BT549 and MCF-7 cells were assessed by Western blotting. Left panels: the levels of STAT6, cGAS, STING, and IRF3. Right panels: p-STING and p-IRF3. The experiments were repeated three times. Data are presented as mean ± SD. **(D)** The expression of autophagy-related proteins in the MDA-231, BT-549 and MCF-7 cells. Left panels: The cells were subjected to immunofluorescence staining of SQSTM1 or LC3. Right panels: Total cell proteins were extracted, and LAMP2, SQSTM1, DNase II and LC3-II were measured by Western blotting. The experiments were repeated three times. Data are presented as mean ± SD. **(E)** The analysis of autophagic flow in the BT-549 cells. The BT-549 cells were treated with 50 μmol/L CQ for 4 hours or 10 nmol/L bafilomycin A1 for 24 hours or with the indicated concentrations of CQ for 4 hours, and total cell proteins were extracted and analyzed by Western blotting. Left panels: The levels of LAMP2 and LC3-II in the bafilomycin A1-treated cells. Middle panels: The levels of SQSTM1 and LC3-II in the CQ-treated cells. Right panels: The levels of DNase II in the indicated concentrations of CQ-treated cells. The experiments were repeated three times. Data are presented as mean ± SD. **(F)** The activity of lysosomes in the MDA-231 and BT-549 cells. After treatment with 10 nmol/L bafilomycin A1 BA1) for 24 hours, the cells were stained with LysoTracker staining kit Solarbio, Beijing, China) according to the manufacturer’s instructions. The experiments were repeated three times. **(G)** Electron microscopy of the autophagic activity in the MDA-231 and BT-549 cells. After treatment with bafilomycin A1 BA1) for 32 hours, the cells were subjected to transmission electron microscopy. The double-membrane enclosed autophagosomes or autolysosomes are labelled by stars. The experiments were repeated twice. β-actin was used as an internal standard in Western blotting. The level of statistical significance was <0.05 *), <0.01 **), < 0.001 ***) and < 0.0001 ****). The images are representative of repeated experiments.

Recently, it has been revealed that MN formed in MDA-231 cells could trigger SASP activity to promote metastasis and progression (15). Like MDA-231, the BT-549 cells are also a triple negative breast cancer (21), and beyond we noted that the BT-549 cells usually present frequent spontaneous formation of MN. The fact provoked our interest about relationship between MN and SASP activity. We used the MDA-231 and BT-549 as well as MCF-7 cells, another classis type of breast cancer cell line (22), for analysis of spontaneous MN formation by immunofluorescent staining of Lamin B1 and NAT10 as described previously (18). The rate of spontaneous MNs in the MCF-7 cells was approximately 5%, that in the MDA-231 cells was approximately 15%, and that in the BT-549 cells was approximately 35% (**Figure 1A**). However, the MNs formed in three cell lines generally contained DNA and Lamin B1 or A/C, nuclear lamina proteins, and other nuclear envelope components, including LBR (Lamin B receptor), nuclear pore complex components (MAB414, nucleoporin 153), the nuclear basket protein TPR (translocated promoter region), and integral membrane components (Sun2 and nesprin2), suggesting that the nuclear membrane of MNs generally maintained structural components similar to the membrane of the main nuclei (**Supplementary Figure 1A**).

MNs are considered a major source of free DNA, which triggers the activation of the SASP through DNA binding to cGAS-STING pathway components (6). Therefore, the MDA-231, BT-549, and MCF-7 cells were analyzed by SA-β-gal staining, and the results indicated that the MCF-7 and MDA-231 cells showed 4% and 8% SA-β-gal positivity, while the BT-549 cells unexpectedly showed no SA-β-gal-positive cells even with repeated staining (**Figure 1B**).

Subsequent Western blotting analysis showed the expression of the STING, pSTING, IRF3, pIRF3, STAT6 in BT-549 cells is lower than that of MDA-231 or MCF-7 cells, indicating that the activity of SASP in BT-549 cell line was significantly lower than that in MDA-231 and MCF-7 cells (**Figure 1C**). The SA-β-gal staining also showed that after treatment with the STING antagonist H-151 (2 μmol/L) for 24 hours, the SA-β-gal positive cells markedly decreased, confirming that the SASP phenotype is mediated *via* cGAS-STING in the MDA-231 cells (**Supplementary Figure 1B**). However, treatment with the STING agonist cGAMP (1) and transfection with Flag-cGAS or Flag-STING did not induce SA-β-gal staining, indicating blockade of cGAS-STING signaling in the BT-549 cells.

Recently, the cGAS-STING pathway was shown to mediate DNA autophagy. Therefore, autophagic activity was first compared among the MCF-7, MDA-231 and BT-549 cells. Western blotting showed that the BT-549 cells presented high expression of LC3-II**,** SQSTM1, and LAMP2, which was not obvious in the MDA-MB-231 or MCF-7 cells, and notably, a high level of DNase II was detected in the BT-549 cells (**Figure 1D**). The BT-549 cells were treated with the autophagic inhibitors CQ (10 μmol/L) for 4 hours or bafilomycin A1 (10 nmol/L) for 24 hours, and the levels of LC3-II, SQSTM1 and LAMP2 were strongly increased, (**Figure 1E**), while not for MCF-7, MDA-231 cells under the treatment of CQ (10 μmol/L) (**Supplementary Figure 1C**), confirming the autophagic activity in the BT-549 cells. Moreover, the expression of DNase II was dose-dependently enhanced by CQ treatment (10 μmol/L) in the BT-549 cells (**Figure 1E**). Furthermore, the staining of LysoTracker, a fluorescent probe for lysosome (23), showed that lysosomes were more abundant in the BT-549 cells than in the MDA-231 cells (**Figure 1F**). The results suggested the involvement of DNA autophagy in the BT-549 cells.

To further confirm the autophagic activity, we also performed electron microscopy of the MDA-MB-231 and BT-549 cells after treatment with bafilomycin A1 (10 nmol/L) for 32 hours, and the results showed that there were many autophagic vesicles in the BT-549 cells but not the MDA-MB-231 cells (**Figure 1G**).

Therefore, the above results demonstrated that breast cancer cells with MNs could differentially present SASP and autophagy, and cells with a high frequency of MNs presented an autophagic phenotype.

### Detection of Genomic DNA Autophagy in the Cytoplasm of Breast Cancer Cells

**Figure 2:**
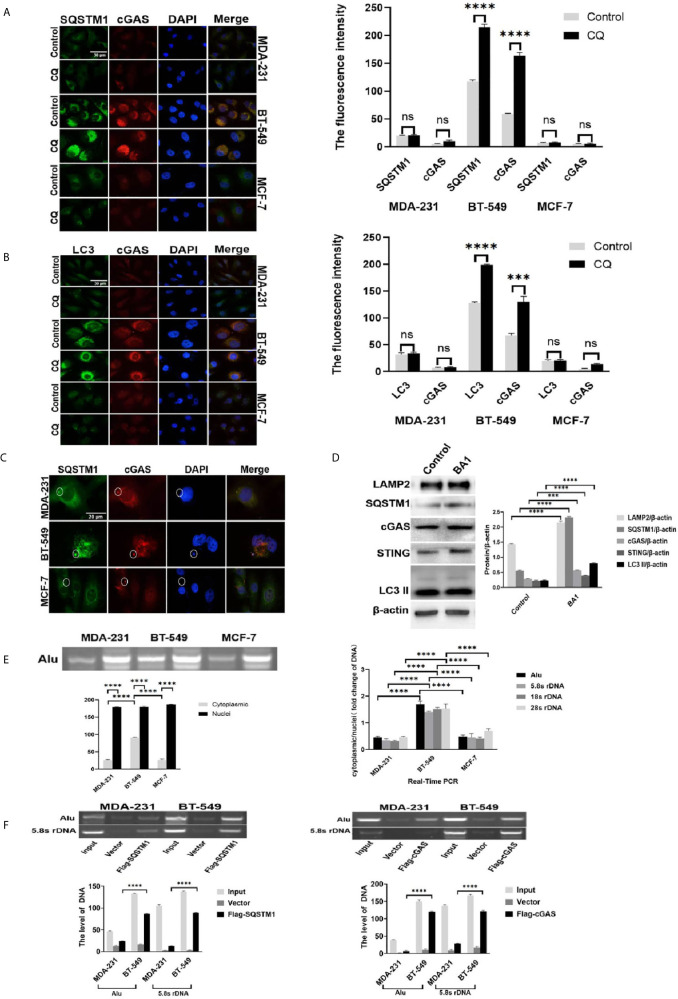
Detection of genomic DNA autophagy in the cytoplasm of breast cancer cells. **(A)** The effects of autophagic inhibition on the expression and distribution of SQSTM1 and cGAS in breast cancer cells. MDA-231 and BT-549 cells were treated with CQ 10 μmol/L) for 4 hours and subjected to double immunofluorescence staining of SQSTM1 green) and cGAS red). The experiments were repeated three times. Data are presented as mean ± SD. **(B)** The effects of autophagic inhibition on the expression and distribution of LC3 and cGAS in breast cancer cells. The MDA-231 and BT-549 cells were treated with CQ 10 μmol/L) for 4 hours and subjected to double immunofluorescence staining for LC3 green) and cGAS red). The experiments were repeated three times. Data are presented as mean ± SD. **(C)** Detection of SQSTM1 and cGAS in the MNs of breast cancer cells. The MDA-231, BT-549 and MCF-7 cells were doubly stained for SQSTM1 green) and cGAS red) by immunofluorescence, and MN colocalization is labeled with circles. The experiments were repeated three times. **(D)** Autophagic inhibition increased the level of cGAS in the BT-549 cells. The BT-549 cells were treated with bafilomycin A1 BA1, 10 nmol/L) for 24 hours, and the levels of cGAS, SQSTM1, STING, LAMP2, and LC3-II were measured by Western blotting. Data are presented as mean ± SD. **(E)** Analysis of cytoplasmic DNA in breast cancer cells. The cytoplasmic or nuclear DNA of the MDA-231, BT-549 and MCF-7 cells was independently isolated, and Alu and rDNA 5.8S, 18S and 28S) were individually detected by PCR from cytoplasmic or nuclear DNA, respectively, as described in the Materials and Methods. Left panels: The amplified Alu products are displayed by gel electrophoresis. The experiment repeated three times. Data are presented as mean ± SD. Right panel: The summarized results of rDNA 5.8S, 18S and 28S) in real-time PCR. The experiments were repeated three times. Results are expressed as mean ± SEM. **(F)** Immunoprecipitation analysis of genomic DNA and cGAS or SQSTM1 in the cytoplasm. The BT-549 cells and the MDA-231 cells were transfected with Flag-cGAS or Flag-SQSTM1 or vector) for 36 hours. After fixation with 1% formalin for 10 minutes, cytoplasmic extracts were isolated as described in the Materials and Methods. The genomic DNA was measured by PCR amplification of Alu- and 5.8S rDNA. Left panels: Flag-SQSTM1. Right panels: Flag-cGAS. β-actin was used as an internal standard in Western blotting. The experiments were repeated three times. Data are presented as mean ± SD. The level of statistical significance was <0.001 ***) and < 0.0001 ****). ns, no significance. All experiments were repeated three times independently, and the images are representative of repeated experiments.

High autophagic activity and frequent MN formation, as well as DNase II expression, indicated the possibility of active DNA autophagy in the BT-549 cells. First, SQSTM1 and LC3, the core proteins of autophagy, were stained by immunofluorescence in the MCF-7, MDA-231 and BT-549 cells. The results showed that the BT-549 cells presented marked cytoplasmic SQSTM1-positive granules or vesicles (~60% cells) (**Figure 2A**), but only a few cells showed cytoplasmic staining for SQSTM1 in MCF-7 and MDA-231. All three kinds of cells presented SQSTM1 positive in occasional Lamin B1-outlined MNs (~5% MNs) (**Supplementary Figure 2A**). Similarly, the BT-549 cells also presented much more cytoplasmic LC3-positive vesicles than the MDA-231 or MCF-7 cells (**Figures 2A, B**). However, almost no LC3 was detected in the MNs of the three kinds of cells (**Supplementary Figure 2B**).

Interestingly, the distribution of cGAS was shown to be similar to that of SQSTM1 or LC3. The BT-549 cells presented accumulation of cytoplasmic cGAS granules that colocalized with SQSTM1 (~50%) and cytoplasmic LC3 (~40% cells), while the MCF-7 and MDA-231 cells were faintly stained for cGAS (**Figures 2A, B**). All three kinds of cells showed staining for cGAS in occasional MNs (3%-5%) (**Supplementary Figure 2C**). In addition, only a few cGAS-positive MNs (~3%) were positive for SQSTM1 but not LC3 (**Figures 2B, C**). STING staining showed that a few cytoplasmic puncta were detected in the MCF-7 and MDA-231 cells but were distributed in the Golgi apparatus in the BT-549 cells (~30%), while the staining was generally weak (see **Supplementary Figure 3C**).

Further, Beclin-1, another autophagy mediator, was stained in breast cancer cells, but few cytoplasmic positive vesicles were detected and only occasional staining in cGAS positive MN (**Supplementary Figures 2D, E**).

To further confirm the autophagic activity, we treated the MDA-MB-231 and BT-549 cells with CQ (10 μmol/L) or bafilomycin A1 (10 nmol/L) for 24 hours, and the results showed that the BT-549 cells presented marked increases in cytoplasmic cGAS, SQSTM1 and LC3, while the MDA-231 and MCF-7 cells only showed slight increases in a few cells (**Figures 2A, B**). However, the MNs in the three kinds of cells presented no significant increase in positive staining of cGAS, SQSTM1, LC3 or Beclin-1 (data not shown). As expected, treatment with bafilomycin A1 markedly increased not only the levels of SQSTM1, LC3-II, and LAMP2 but also cGAS and STING in the BT-549 cells (**Figure 2D**).

The colocalization and accumulation of SQSTM1, cGAS and LC3 in the cytoplasm suggested the possibility of selective cytoplasmic DNA autophagy. To directly verify the genomic DNA in the cytoplasm, we independently isolated cytoplasmic DNA from the MCF-7, MDA-231 and BT-549 cells and the existence of genomic DNA was determined by PCR detection of Alu- or rDNA (5.8S, 18S and 28S) repeated sequences. The gel electrophoresis results showed that the cytoplasmic abundance of Alu in the BT-549 cells was significantly higher than that in the MDA-231 and MCF-7 cells (**Figure 2E**). Moreover, real-time PCR confirmed the greater cytoplasmic abundance of Alu and rDNA (5.8S, 18S and 28S) in the BT-549 cells than in the MCF-7 and MDA-231 cells (**Figure 2E**).

To explore the potential interaction between cGAS or SQSTM1 and genomic DNA in the cytoplasm, co-immunoprecipitation was carried out. Flag-SQSTM1 and Flag-cGAS were transfected into the MDA-231 and BT-549 cells, and the cytoplasmic fractions of Flag-SQSTM1 and Flag-cGAS were immunoprecipitated. The sequences of Alu- or 5.8S rDNA could be detected in either precipitated Flag-SQSTM1 or Flag-cGAS and were more abundantly detected in the BT-549 cells than in the MDA-231 cells (**Figure 2F**). Moreover, the sequences of Alu DNA in the cytoplasm showed greater detection in the BT-549 cells than in the MDA-231 cells by FISH, and following treatment with CQ and bafilomycin A1, the cytoplasmic signals were enhanced in the BT-549 cells (**Supplementary Figure 2G**), but the level of the LaminA/C or LBR in all three kind of cells were not changed in the presence of CQ (50 μmol/L) for 36 hours, (**Supplementary Figure 2F**).

Taken together, these data indicated that DNA autophagy in breast cancer cells could be selective autophagy of cytoplasmic free DNA but not nucleophagy and possibly involved cGAS, SQSTM1 and LC3.

### DNA Autophagy in the Cytoplasm Was Involved in the Coordination of cGAS and SQSTM1

**Figure 3:**
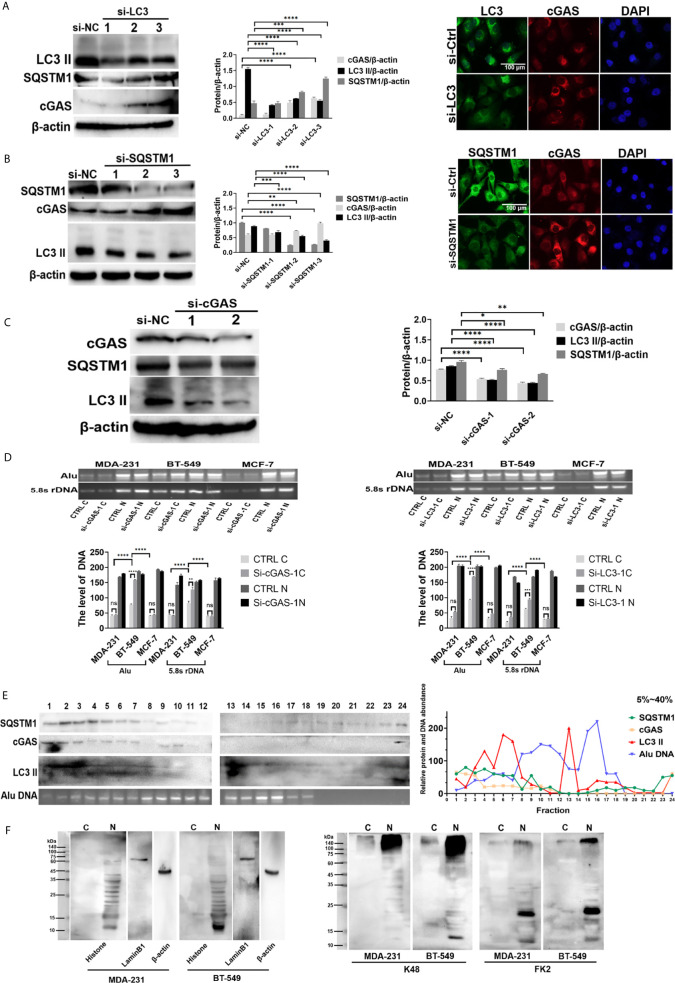
DNA autophagy in the cytoplasm was involved in the coordination of cGAS and SQSTM1. **(A)** The depletion of *LC3* increased the level of cGAS. After knockdown by *LC3* siRNA 1, 2, 3 in BT-549 cells, the level of cGAS was measured by Western blotting left panels), while the cellular distribution of LC3 (green) and cGAS (red) in the *LC3* siRNA 3-transfected cells was analyzed by immunofluorescence staining right panels). siNC: control iRNA. The experiments were repeated three times. Data are presented as mean ± SD. **(B)** The depletion of *SQSTM1* increased the level of cGAS but decreased LC3-II. After knockdown of *SQSTM1* by siRNA 1, 2, 3) in BT-549 cells, the levels of cGAS or LC3-II were measured by Western blotting left panels), and the c ellular distribution of cGAS and LC3 in the *SQSTM1* siRNA 3-transfected cells was analyzed by immunofluorescence staining right panels). siNC: control iRNA. The experiment repeated three times. Data are presented as mean ± SD. **(C)** The depletion of *cGAS* decreased the level of LC3-II. After knockdown by *cGAS* siRNA 1, 2 in BT-549 cells, the levels of LC3-II or SQSTM1 were measured by Western blotting. siNC: control iRNA. The experiment repeated three times. Data are presented as mean ± SD. **(D)** The silencing of autophagic genes increased the level of cytoplasmic DNA. Cytoplasmic or nuclear DNA was extracted from the MCF-7, MDA-231 and BT-549 cells or their corresponding cells with knockdown of *SQSTM1* or *LC3* by siRNA. The levels of cytoplasmic C) or nuclear N) DNA were measured by PCR amplification of Alu and 5.8S rDNA sequences, and the quantitative analysis was performed with the results of gel electrophoresis. The experiments were repeated three times. Data are presented as mean ± SD. **(E)** Sucrose density gradient analysis of the cytoplasmic fractions of the BT-549 cells. Cytoplasmic extracts from the BT-549 cells were isolated and fractioned through sucrose gradient centrifugation, and SQSTM1, cGAS, and LC3 II in each fraction were measured by Western blotting. Cytoplasmic genomic DNA in selected fractions was analyzed by Alu-based PCR amplification and gel electrophoresis. The amounts of the indicated proteins were quantified using ImageJ and plotted. The experiments were repeated three times. **(F)** Analysis of cytoplasmic histones and ubiquitination in breast cancer cells. Cytoplasmic or nuclear extracts from the MDA-231 or BT-549 cells were independently isolated by acid extraction and analyzed by Western blotting. Left panels: Core histone; middle panels: K48 antibody detection; right panels: FK2 antibody detection of mono- or polyubiquitinated proteins. The arrows indicate the putative histones. C, cytoplasmic; N, nuclear. The level of statistical significance was <0.05 *), <0.01 **), < 0.001 ***) and < 0.0001 ****). ns, no significance. The experiments were repeated three times, and the images are representative of repeated experiments.

To explore the autophagic flux in DNA autophagy, we investigated the relationship between cGAS and SQSTM1 or LC3. After knockdown of either *LC3* or *SQSTM1* by siRNA, the level of cGAS was obviously increased in the BT-549 cells, as shown by Western blotting (**Figures 3A, B**, left panels), and in immunofluorescent staining (**Figures 3A, B**, right panels). Moreover, depletion of *LC3* increased the SQSTM1 levels (**Figure 3A**, left panels). In contrast, after knockdown of *cGAS* by siRNA, the levels of either LC3-II or SQSTM1 were decreased in the BT-549 cells, as shown by immunofluorescence and Western blotting (**Figure 3C**).

After knockdown of *cGAS* by interfering RNAs, gel electrophoresis showed that the levels of cytoplasmic Alu- and rDNA sequences increased obviously in the BT-549 cell lines but not in the MCF-7 or MDA-231 cell lines (**Figure 3D**). Similarly, knockdown of *LC3* also resulted in the same findings (**Figure 3D**).

To explore the potential autophagic complex of SQSTM1, we further analyzed cGAS, LC3 and free DNA through cytoplasmic fractioning in a density gradient fraction assay, in which the detection of SQSTM1 partly overlapped in fractions containing cGAS, LC3 and free DNA (**Figure 3E**). Moreover, a coprecipitation analysis showed that genomic DNA could be detected in the MDA-231 and BT-549 cells transfected with either Flag-SQSTM1 or Flag-cGAS (**Figure 2F**). In addition, endogenous cGAS in the BT-549 cells coprecipitated with transfected Flag-SQSTM1 (**Supplementary Figure 3A**, upper panel).

However, so far there are no evidences on the direct interaction of SQSTM1 with either cGAS or DNA, and how SQSTM1 participates in DNA autophagy needs to be addressed. Generally, SQSTM1 recognizes ubiquitinated substances during autophagy, and it has been reported that cGAS undergoes K48-linked ubiquitination at K414, leading to SQSTM1-dependent selective autophagic degradation (24). Thus, Flag-SQSTM1 was transfected into the BT-549 cells, but K48-ubiquitinated cGAS could not be detected by Western blotting (**Supplementary Figure 3A**, lower panel).

Since DNA is usually coated with histones or other chromatin-binding proteins, cytoplasmic free DNA was also assumed to be bound to histones. To clarify this, we isolated cytoplasmic histones from the BT-549 or MDA-231 cells by acid-based extraction (20). Western blotting showed that cytoplasmic histones could be detected in both the BT-549 and MDA-231 cells, but the BT-549 cells presented more cytoplasmic histones than the MDA-231 cells (**Figure 3F**); these structures could be detected by anti-K48 ubiquitin and FK2 antibodies (against poly- or monoubiquitinated proteins) (**Figure 3F**). Moreover, immunofluorescence staining showed that more intensive staining of FK2 could be detected in the cytoplasm of the BT-549 cells than in that of the MDA-231 cells (**Supplementary Figure 3B**).

Taken together, the results suggested that free cytoplasmic DNA autophagy could be mediated in a complicated process, assumedly involving cGAS binding to DNA and recognition of ubiquitinated histones by SQSTM1, respectively.

### Genomic DNA in the Cytoplasm Could be Derived From Either Damaged Nuclei or MNs

**Figure 4:**
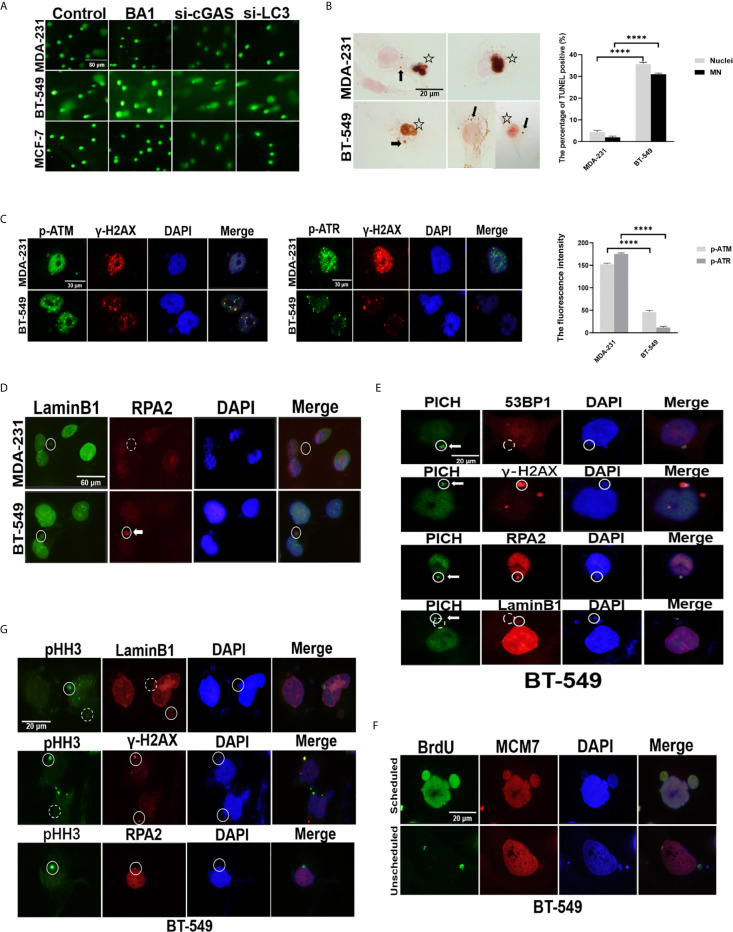
Genomic DNA in the cytoplasm could be derived from either damaged nuclei or MNs. **(A)** Comet assays of the status of DNA damage in breast cancer cells. The original MDA-231, BT-549 and MCF-7 cells, those in the presence of 10 nmol/L BAI bafilomycin A1) for 24 hours, or cells with silencing of cGAS or LC3 by transfection with either si-cGAS or si-LC3 for 48 hours were subjected to comet assays. Their more detailed data are summarized in **Table 1**. The experiments were repeated three times. **(B)** TUNEL analysis of breast cancer cells. A TUNEL assay was carried out on the MDA-231 and BT-549 cells, and positive staining in the nucleus and/or MNs was evaluated in whole slides and is presented as percentages. The experiments were repeated three times. Data are presented as mean ± SEM. Arrows indicate positive MNs, while stars label positive nuclei. **(C)** The status of DNA damage in breast cancer cells. The MDA-231 and BT-549 cells were stained for γ-H2AX and p-ATM or p-ATR, respectively left panels). The staining intensity was evaluated and plotted right panel). The experiments were repeated three times. Data are presented as mean ± SD. **(D)** Double staining of RPA2 and Lamin B1 was performed in the MDA-231 and BT-549 cells. Solid circles label positive staining, and dashed circles indicate weak or negative staining. Arrow indicates RPA2 foci in MNs. The experiments were repeated three times. **(E)** Double staining of PICH and RPA2 or 53BP-1 or γ-H2AX or Lamin B1 was performed in the BT-549 cells. Solid circles indicate positive staining, and dashed circles indicate weak or negative staining. Arrows indicate PICH foci in MNs. The experiments were repeated three times. **(F)** BrdU incorporation assays. After incubation with BrdU for 30 minutes, the BT-549 cells were doubly stained with BrdU and MCM7. Arrows indicate unscheduled replication in MNs. **(G)** Analysis of DNA damage in the MNs in breast cancers. Double staining of Lamin B1, RPA2 or γ-H2AX and pHH3 was performed in the BT-549 cells. Solid circles indicate highly stained MNs, and dashed circles indicate negative or weak staining of MNs. The experiment repeated three times. The level of statistical significance was < 0.0001 ****). The images are representative of repeated experiments.

Since the cytoplasmic DNA undergoing autophagy was genomic DNA from the nuclei and nuclear membrane in the breast cancer cells were not generally broken (**Supplementary Figure 1A**), DNA damage was assumed to be involved. The level of DNA damage in the BT-549, MCF-7 and MDA-231 cells was analyzed by comet assays. **Table 1** shows that the tail length of the BT-549 cells was substantially longer than that of the MDA-231 (76.83, P<0.0001) and MCF-7 cells (70.59, P<0.0001), the tail intensity of the BT-549 cells was greater than that of the MDA-231 (70319, P<0.01), and MCF-7 cells (75513, P<0.0001), and the tail movement of the BT-549 cells was also obviously higher than that of the MDA-231 (13.09, P<0.0001) and MCF-7 cells (15.66, P<0.0001). These results indicated that the DNA damage was the most severe in the BT-549 cells (**Table 1** and **Figure 4A**).

**Table 1:** The results of the Comet assay.

Cell lines		Tail length		Tail intensity		Tail movement	
		Mean	SD	Mean	SD	Mean	SD
MDA-231	Control	76.83	45.00	70319.47	59133.3	13.09	14.16
	Bafilomycin A1	87.43	43.28	69149.58	61988.6	16.14	20.33
	NC	78.88	46.56	69820.57	59621.2	12.78	13.21
	si-LC3	66.26	37.16	94829.15	116894	19.06	28.14
	si-cGAS	74.13	61.62	72505.77	72552.6	13.19	11.86
BT-549	Control	109.94(****)	39.37	185572.14(****)	139055	42.63(****)	25.33
	Bafilomycin A1	182.468(****)	71.71	321391.656(***)	183954	60.123(*)	51.03
	NC	100.67	45.72	189102.12	139234	45.22	25.43
	si-LC3	190.637(****)	81.25	484393.68(****)	136164	95.130(**)	35.25
	si-cGAS	163.324(****)	68.48	392065.161(***)	111780	70.662(**)	47.03
MCF-7	Control	70.59	41.03	75513.63	102705	15.66	21.45
	Bafilomycin A1	69.48	38.46	84516.05	114563	15.93	20.43
	NC	68.23	40.92	79333.41	99012.3	16.01	21.22
	si-LC3	59.94	30.88	95231.169(*)	100438	15.09	21.39
	si-cGAS	77.88	55.12	87188.13	93316.8	15.54	21.37

Results are expressed as mean ± SD. (*p ＜ 0.05, **p ＜ 0.01, ***p ＜ 0.001, ****p ＜ 0.0001).

Following bafilomycin A1 treatment, the length, intensity and movement of the tails in the BT-549 cells, but not in the MCF-7 and MDA-231 cells, increased significantly (182.47 (P=0.0008), 321391.66 (P=0.0002), and 60.12 (P=0.0024), respectively) (**Table 1** and **Figure 4A**). Similarly, knocking down *cGAS* and *LC3* in BT-549 cells by siRNA also increased the tail values in the comet assay (**Table 1**, P<0.001) (**Table 1** and **Figure 4A**). These results indicated that inhibition of autophagy could influence the status of DNA damage.

Moreover, TUNEL assays were performed to directly measure DNA breaks in breast cancer cells. BT-549 cells presented more TUNEL-positive cells than those of MCF-7, MDA-231, and the percentage of TUNEL-positive BT-549 cells was approximately 35%, and the percentage of MDA-231 cells was 5% (P<0.05) (**Figure 4B**). Furthermore, the expression of ATM, ATR, and γ-H2AX in the MDA-231 and BT-549 cells further confirmed that the BT-549 cells generally had lower levels of ATM, ATR, and γ-H2AX, indicating a failure in the DNA damage response (**Figure 4C**).

Interestingly, the TUNEL assay also showed that the BT-549 cells presented TUNEL positivity not only in the nuclei but also in 30% of the MNs (**Figure 4B**), while there were no differences in 53BP1 or γ-H2AX staining in the MNs among the BT-549, MDA-231 and/or MCF-7 cells (**Supplementary Figure 4A**). However, in further DNA damage analysis, RPA2 (replication protein A2) and PICH (PLK1-interacting checkpoint helicase), the factors involved in DNA replication, were stained in breast cancer cells. Some MNs in the BT-549 cells, but not MDA-231 and/or MCF-7 cells, exhibited bright foci with RPA2 or PICH staining (**Figures 4D, E**), indicating DNA single or double breaks owing to DNA replication. Some MNs could be separately labeled by BrdU, indicating unscheduled DNA replication in the MNs (**Figure 4F**). More interestingly, immunofluorescence staining of pHH3 (phosphorylated histone 3), a marker of chromatin condensation, could be detected in some MNs, especially in the BT-549 cells, similar to mitotic cells, and pHH3-stained MNs were usually Lamin B1 negative (**Figure 4G**), which indicated that this DNA damage might be caused by a process similar to apoptosis in the MNs (25). The results indicated that at least a portion of cytoplasmic DNA possibly came from the collapse or degradation of MNs.

Taken together, the results suggested that free cytoplasmic DNA was involved in the DNA damage response failure and its consequential MN formation, part of which underwent collapse owing to replication and DNA damage.

### Inhibition of DNA Autophagy Induced Growth Arrest or Cell Death of Cancer Cells

The above results demonstrated that breast cancer cells presented high DNA autophagic activity, which raised the question of whether autophagy influences the biological activity of this kind of cancer cell.

**Figure 5:**
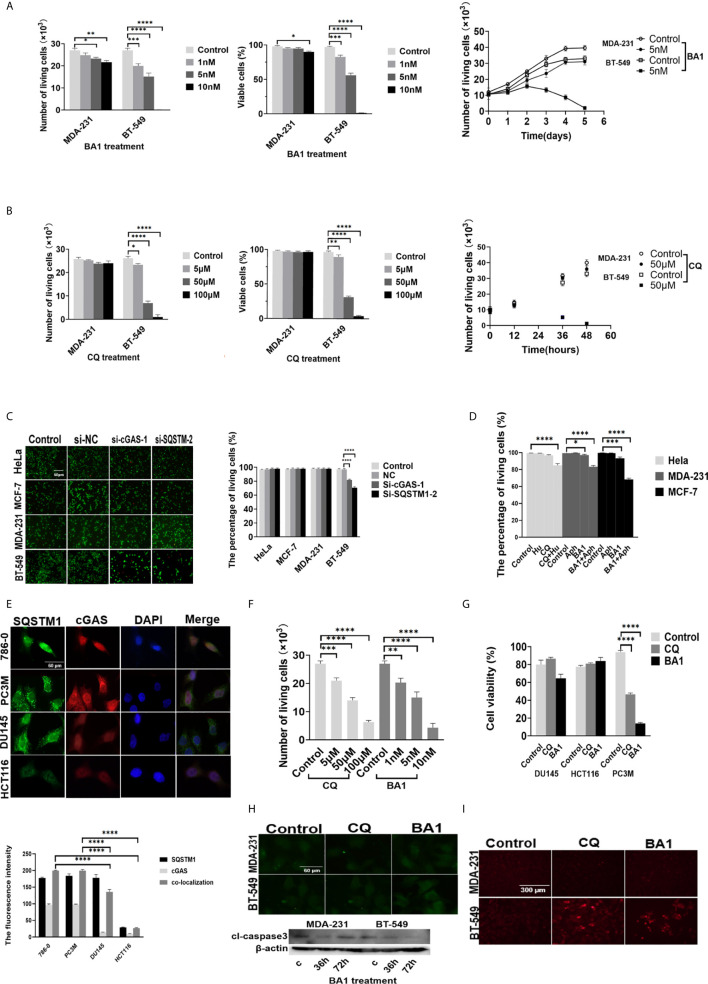
Inhibition of DNA autophagy induced growth arrest or cell death of cancer cells. **(A, B)** The effects of autophagic inhibition on the survival and growth of breast cancer cells. Left: MDA-231 and BT-549 cells were treated with the indicated concentrations of BA1 bafilomycin A1) or CQ for 36 hours, and cell viability was measured. Right: The growth of the MDA-231 and BT-549 cells was analyzed in the presence of 10 nmol/L BA1 or 50 μmol/L CQ for the indicated days. Cell viability was measured by Trypan blue exclusion assays. **(C)** The effects of downregulation of either cGAS or SQSTM1 on the survival of cancer cells. The MDA-231, BT-549, MCF-7 and HeLa cells were transfected with si-cGAS and si-SQSTM1 for 48 hours, and the post-transfection cell viability was analyzed by live and dead assays. **(D)** Enhancement of DNA damage sensitized cancer cells to autophagic inhibitors. The MCF-7, MDA-231, and HeLa cells were treated with hydroxyurea 0.5 mmol/L) or aphidicolin 1 μmol/L) for 4 hours and then further incubated in the presence of CQ 50 μmol/L) or bafilomycin A1 10 nmol/L) for 36 hours. Cell viability was analyzed by live and dead assays. **(E)** Upper panel: Double staining of SQSTM1 green) and cGAS red) was performed in the 786-0, HCT116, DU145 and PC3M cells. Lower panel: The quantification of immunofluorescence co-localization in three cells. **(F)** The 786-0 cells were treated with the indicated concentrations of CQ or BA1 for 36 hours. Cell viability was measured by live and dead assays. **(G)** The effects of autophagic inhibition on the survival of cancer cells. The HCT116, DU145 and PC3M cells were treated with CQ 50 μmol/L) or bafilomycin A1 BA110 nmol/L) for 36 hours, and cell viability was measured by MTS assays. **(H)** Detection of the active form of caspase-3 in the autophagy-inhibited breast cancer cells. The MDA-231 and BT-549 cells were treated with either CQ 50 μmol/L) or BA1 bafilomycin A1, 10 nmol/L) for 36 hours, stained for cl-caspase3 by immunofluorescence upper) and measured by Western blotting lower). **(I)** The inhibition of autophagy induced caspase-independent cell death in the BT-549 cells. After treatment with either CQ 50 μmol/L) or BA1 bafilomycin A1, 10 nmol/L) for 36 hours, the BT-549 cells were incubated *in vivo* with dihydroethidium 1 mmol/L) for 30 minutes, and ethidium-stained cells were counted in a total of 500 cells. The images are representative of repeated experiments. The level of statistical significance was <0.05*), < 0.01 **), < 0.001 ***), and < 0.0001 ****). Each of experiments was performed in triplicate, and all experiments were independently repeated at least three times. The images are representative of repeated experiments. Results are expressed as mean ± SEM.

To further determine the role of autophagy in breast cancer cells, we grew MCF-7, MDA -231 and BT-549 cells in the presence of bafilomycin A1 at various concentrations (0 nmol/L, 1 nmol/L, 5 nmol/L, 10 nmol/L) for 72 hours, and the results showed that the growth of both the MDA-231 and BT-549 cells was inhibited in a dose-dependent manner (**Figure 5A**). However, the BT-549 cells showed cytotoxicity in the presence of 10 nmol/L bafilomycin A1. This finding was confirmed by time course analysis, in which the BT-549 cells with high autophagic activity died after treatment with 10 nmol/L bafilomycin A1 for 72 hours, while the MDA-231 cells only showed growth inhibition (**Figure 5A**).

Similarly, after further treatment of the MCF-7, MDA-231 and BT-549 cells with different concentrations of CQ (0 μmol/L, 5 μmol/L, 50 μmol/L, 100 μmol/L) for 72 hours, cell survival and growth were evaluated (**Figure 5B**).

The effects of cGAS or SQSTM1 expression on cell growth and survival of breast cancer cells were also clarified through silencing of either cGAS or SQSTM1 in the MCF-7, MDA-231 and BT-549 cells. The viability of the BT-549 cells was markedly reduced after either si-cGAS or si-SQSTM1 silencing. However, the MCF-7, MDA-231 and HeLa cells were not affected significantly (**Figure 5C**). Similarly, the BT-549 cells, but not MDA-231 cells, were inhibited by treatment with the STING antagonist H-151 (2 μmol/L, 10 μmol/L, 20 μmol/L) for 24 hours (**Supplementary Figure 5A**).

To further clarify inhibition of autophagy to DNA autophagy under enhanced DNA damage, MCF-7, MDA-231, HeLa cells treated with Hydroxyurea (26) (0.5mmol/L) or Aphidicolin (27) (1μmol/L), which could not only induce cytoplasmic accumulation of cGAS/SQSTM1, but also became sensitive to the inhibitors of CQ (50 μmol/L) or bafilomycinA1 (10 nmol/L) (**Figure 5D** and **Supplementary Figures 5B–D**).

To further expand the observation of DNA autophagic inhibition to cell activity, we screened a series of human cancer cells with immunofluorescence double-staining of cGAS and SQSTM1, and HCT116, 786-0, PC3M, and DU145 cells were screened. Three categories of cancer cells—cells with high cGAS and SQSTM1 (786-0 and PC3M), low cGAS and SQSTM1 (HeLa and HCT116), and high SQSTM1 but low cGAS (DU145)—were selected (**Figure 5E**). In further analysis, the survival of 786-0 cells with strong cGAS and SQSTM1 expression was reversely to the treatment of CQ or bafilomycin A1 in a dose-dependent way (**Figure 5F**), and the PC3M cells were also reduced significantly in the treatment of CQ (50 μmol/L) or bafilomycin A1 (10 nmol/L) (**Figure 5G**). In contrast, HCT116 cells with low levels of cGAS and SQSTM1 were not significantly affected with the treatment of CQ (50 μmol/L) or bafilomycin A1 (10 nmol/L) (**Figure 5G**). In similar, DU145 cells with high SQSTM1 but low cGAS levels were not affected in above treatments (**Figure 5G**). The results implied that the growth or survival of cancer cells with high DNA autophagy was sensitive to autophagic inhibition.

To explore the cell death induced by DNA autophagic inhibition, we analyzed the CQ- or bafilomycin A1-treated MCF-7, MDA-231 and BT-549 cells, and the active form of caspase-3 was not detected by staining (**Figure 5H**). Therefore, caspase-independent cell death (CICD) was investigated. Autophagy-related CICD could be achieved through lysosomal membrane permeabilization (LMP), but because CQ is an LMP inducer and bafilomycin A1 is an LMP inhibitor, LMP-induced cell death could be ruled out. Alternatively, CICD is usually mediated by increasing ROS (reactive oxygen species) owing to mitochondrial outer-membrane permeabilization (MOMP). The production of ROS was measured in the presence of CQ and bafilomycin A1 by dihydroethidium (1 mmol/L), and the results showed that either CQ or bafilomycin A1 could increase ethidium-stained BT-549 cells but only slightly affected the MDA-231 or MCF-7 cells (**Figure 5I**), confirming that CQ or bafilomycin A1 treatment could induce caspase-independent cell death in the BT-549 cells.

The results demonstrated that DNA autophagy could be necessary for the survival of cancer cells by clearing cytoplasmic free DNA to protect against cell death.

## Discussion

Autophagy is an important adaptive process to recycle substances or clear damaged organelles. For decades, autophagy has been thoroughly elucidated in terms of its process, forms, regulation and biological roles (28). DNA autophagy has also been identified, especially as a mechanism against exogenous invasion of organisms, and is considered an innate immune mechanism (29) However, apparently, unlike that of other substances, autophagy of genomic DNA, the cellular genetic materials, is difficult to be considered for multicellular organisms. Nevertheless, in unicellular lower eukaryotes such as yeasts, DNA autophagy, in which cell nuclei can undergo autophagy by well-established regulatory pathways for nucleophagy through either piecemeal microautophagy of the nucleus (PMN) or late nucleophagy, has been identified (29). Even entire nuclei could be degraded by macroautophagy in filamentous fungi. For mammalian cells, MN-related nucleophagy has been described occasionally, but mechanistically, its detailed process is still elusive. Mammalian cells have a nuclear lamina structure, which is different from that of lower eukaryotes, such as yeasts, with no comparable lamins or a fibrous nuclear envelope scaffold (29). Nevertheless, nucleophagy is considered to play an important role in maintaining cellular genomic stability, detecting DNA damage, and regulating cellular apoptosis, as well as cellular senescence (30, 31). MN assays showed increased MN frequencies in breast cancer lymphocytes, which were correlated with the progression of breast cancer (32). MNs are abnormal components that exists in the cytoplasm, independent of the nuclear nucleus. Autophagy also contributes to the elimination of MN (29). However, for most cancer cells, MNs can persist, arguing the general elimination of MNs by nucleophagy. In addition, similar to other studies, our investigations showed that cGAS and/or SQSTM1 could be detected in some MNs. However, the percentage of MNs with colocalization was low even in the highly abundant MNs in the BT-549 cells. Nucleophagy was not a prevalent event in cancer cells. A previous report showed the interaction between Lamin B1 and LC3 and suggesting that it is a nucleophagic mechanism (25). However, we still did not observe such interactions in our experiments, for instance, in BT-549, MDA-231 or 786-0 cells, all of which usually showed a relatively high frequency of MN formation. Another possibility could not be ruled out: MNs undergoing nucleophagy could directly fuse with lysozyme, but this hypothesis needs to be further explored.

Selective DNA autophagy (33) has been well clarified in mammalian cells. It has been demonstrated that free DNA or RNA could directly mediate microautophagy *via* LAMP2 (lysosome-associated membrane protein 2) without the need for LC3 or other autophagic factors, but the nucleic acid transporter SIDT2 (SID1 transmembrane family member 2, SIDT2) is an integral lysosomal membrane protein for translocation into the lysosomal lumen (34). Nevertheless, specific DNA sensors, such as cGAS-STING, have also been revealed to participate in DNA autophagic initiation. cGAS binds to DNA (in MNs or free) and recruits Beclin-1 and STING, promoting autophagy. cGAS generates cGAMP and stimulates the STING-Golgi apparatus, but some studies have also shown that STING itself could mediate autophagy after its binding to DNA, and the intrinsic domains of STING could directly interact with LC3 (8). In a recent report, exogenous plasmids were first recognized by DAI/ZBP1 (DNA-dependent activator of interferon regulatory factors/Z-DNA binding protein 1) but not cGAS (35). However, in our study, upon introducing genomic DNA into the MCF-7, MDA-231 or BT-549 cells, we found that cytosolic inclusions of various sizes were positive for cGAS, LC3 and lysosomes, confirming DNA autophagy (**Supplementary Figure 6**). However, few inclusions were Beclin-1 positive (**Supplementary Figure 6**). Therefore, it seemed that cytoplasmic DNA autophagy might involve different forms owing to the source of DNA and especially the DNA status and its level, nucleophagy, and selective autophagy. Apparently, the molecular mechanism could be diverse, such as LC3-dependent or LC3-independent DNA sensors. In actual conditions, free DNA is not naked but is instead usually bound to nuclear proteins. Therefore, genomic DNA from MN collapse or nuclear release might trigger a more complicated autophagic reaction to both DNA and proteins, especially ubiquitination (see below).

In recent years, extensive research has demonstrated that SASP is activated by the cGAS-STING pathway, and its proinflammatory role has been demonstrated to be crucial for the occurrence of autoinflammatory disorders, age-related diseases and even cancer progression (8). However, either cGAS or STING alone or their combination could mediate DNA autophagy (8). The described data also indicated that cGAS could play an important role in autophagy. These findings raise an important question of how the decision to choose SASP or autophagy is made in cells. Our research showed that autophagy was usually found in cells with profound DNA damage, while increased DNA damage could induce DNA autophagic activity, indicating that the extent of DNA damage could be a factor influencing this determination. cGAS-mediated SASP and autophagy could respond to DNA damage. Severe DNA damage should be cleared by autophagy, but relatively less severe damage triggers SASP. Apparently, the cellular ability of DNA damage repair could also be a factor. These findings raise the question of whether there is any difference between cGAS in mediating SASP and autophagy. The details of cGAS recognition of DNA to induce autophagy or SASP are still unclear. However, more importantly, released genomic DNA from the cytoplasm is not protein-free but is instead bound with histones or other nuclear proteins (36). The complex of DNA and protein could more easily activate autophagic activity since histones are usually ubiquitinated and generally recognizable by SQSTM1. To date, many studies have observed that SQSTM1 could be detected in the cell nucleus by either tagged SQSTM1 or immunohistochemistry (24). In fact, the nuclear localization signal of SQSTM1 has been revealed, and its nuclear translocation has been demonstrated to be involved in the DNA damage response (37, 38). Therefore, the cytoplasmic or nuclear distribution of SQSTM1 could be similar. Thus, it is more likely that with severe DNA damage or repair failure, a relative amount of genomic DNA with coated proteins is released into the cytoplasm to trigger an autophagy-mediated clearance response.

Recent reports suggest that activation of cGAS upon binding to DNA could trigger activation of STING, leading to either SASP or autophagy, and MCF-7, MDA-231 and BT-549 cells presented different endogenous levels of STING or phosphorylated STING, which was consistent with their SASP and autophagic phenotypes (**Figures 1B, C**). Moreover, endogenous or exogenous STING was present in a few cytoplasmic vesicles in the MCF-7 and MDA-231 cells but was distributed in the Golgi apparatus in the BT-549 cells and could be disrupted by brefeldin A (BFA) (**Supplementary Figure 3C**). In the BT-549 cells, the level of STING markedly increased in the presence of bafilomycin A1 (10 nmol/L) (**Figure 2D**) or with downregulation of *DNase II* (**Supplementary Figure 3D**). However, after treatment of the BT-549 cells with either the STING antagonist H-151 (2 μmol/L) or agonist cGAMP (300 nmol/L) for 24 hours, SQSTM1 or LC3 presented no change in immunofluorescence staining. Thus, degradation of STING may be involved in the autophagic process, while its activity in the regulation of autophagy in BT-549 cells requires further exploration.

Cytoplasmic DNA can easily be derived from mitochondria, organelles in the cytoplasm, but the mechanism by which genomic DNA accumulates in the cytoplasm is still unclear. MN formation is believed to be a major source since various reports have shown that MNs from some cancer cells are not intact in their nuclear lamina due to RB deficiency (39). Indeed, defects in nuclear membrane assembly, either aberrant nuclear pore complexes NPCs) or nuclear lamina defects owing to lamina gene mutations, have been shown to result in nuclear irregularity, lobulation or MN formation to cause cellular senescence and the SASP (6). More than 60% of MNs are disrupted, and then, damaged DNA is released (6, 39) However, in our investigation, as well as a previous report (18), MNs formed in a variety of cancer cell lines, including breast, colorectal, cervical and kidney cancer cell lines, generally had intact nuclear lamina proteins, such as Lamin A/C or B or their receptor LBR, nuclear pore complex Nup153), TPR blanked protein), and integral membrane proteins Sun2, nesprin2) (40). Nuclear rupture did not appear to be a prevalent event for cancer cells, and cytoplasmic DNA from MNs needs to be further explored. Similar to its main nucleus, MNs can undergo various activities, including replication, transcription and DNA damage. Chromothripsis has been demonstrated to be a consequence of DNA damage in MNs (10). This research also showed that unscheduled DNA replication and DNA damage could be detected in a portion of the MNs. Interestingly, some BT-549 cells with abundant MNs more frequently presented condensed focal staining of RPA2 or PICH, both of which participate in the DNA damage response; the former usually binds to single-stranded DNA, and the latter binds to double-stranded DNA (41, 42). More importantly, some MNs were frequently observed in pHH3-positive cells, similar to BT-549 cells (**Figure 4F**). It has been acknowledged that pHH3 is mainly found during chromatin condensation in mitosis and in apoptotic nuclei of cells (43, 44). For determination of whether pHH3-positive MNs undergo mitosis, cells were stained for Hec1 (45), a protein involved in kinetochore assembly. The results proved that no MNs were positive, but only mitotic and apoptotic cells were stained. In a cell-free apoptotic model, nuclear condensation could sequentially proceed with condensation, a nuclear necklace, collapse or disassembly (25, 35). Similarly, pHH3-stained MNs also presented these morphological changes (**Figure 4F**), indicating that MNs could undergo collapse owing to replication and damage. Accordingly, a portion of the MNs in the BT-549 cells showed positive staining of RPA2 and PICH, especially in the foci-staining pattern (**Figures 4D, E**), indicating severe DNA damage in these MNs. Apparently, the more MNs formed, the more frequently breakage was detected. In addition, the release of nuclear eccrDNA extrachromosomal circular rDNA) should be another source of cytoplasm see below).

Since MN formation can be generally induced by a variety of genotoxic agents, DNA damage is reasonably considered a key process, and aberrant mitosis is widely accepted (6, 12). However, recent studies have suggested that for cancer cells, DNA replication stress could be a likely common mechanism (41, 46–48). Oncogenic mutations induce accelerated DNA replication and trigger replication stress. The so-called common fragile sites in the genome, such as rDNA, are difficult to replicate, and stalled or collapsed replication forks usually induce the formation of UFBs ultrafine bridges) or lagging chromosomes to result in MN formation or to generate free DNA fragments, such as eccrDNA, which are hard to enclose in the late phase of mitosis during nuclear membrane assembly and are consequently released to the cytoplasm (16, 42). In our investigation, in addition to MNs, cytoplasmic DNA from the genome, including Alu-repeated sequences and rDNA, was easily detected, indicating a replication stress-related mechanism. Moreover, intra-S phase checkpoints mediated by ATR and ATM kinases are crucial to replication stress, and their deficiency causes replication stress-related DNA damage (49). The BT-549 cells generally had low levels of pATM and pATR and high formation of MNs and cytoplasmic DNA as well as strong TUNEL staining, suggesting relationships between these factors.

Autophagy is a form of cellular activity that adapts to endogenous and environmental changes. Although gene mutations related to the regulation of autophagy have been clarified in tumorigenesis, for some kinds of cancer cells, inhibition of autophagy could promote their growth and survival, indicating that autophagic activity could be necessary for these cancer cell (50). However, how to determine the sensitivity of cancers to autophagic inhibition is still not established. Meanwhile, some studies have shown that the inhibition of autophagy can affect the viability of cancer cells (51). This investigation showed that inhibition of DNA autophagy could decrease cell viability, suggesting its potential therapeutic utility in cancer treatment, especially for cancer cells deficient in DNA repair. In recent years, DNA damage repair deficiency has been successfully used in cancer therapy; for instance, cancers with MSI microsatellite instability) can be treated with immune checkpoint blockade-based immunotherapies, while genomic mutations of BRCA1/2 or HRR homologous recombination repair) are targets of PARP inhibitors. DDR deficiency has been considered a promising anticancer target (52–54) Targeting autophagy could be another approach to treat DDR-deficient cancers.

**Figure 6:**
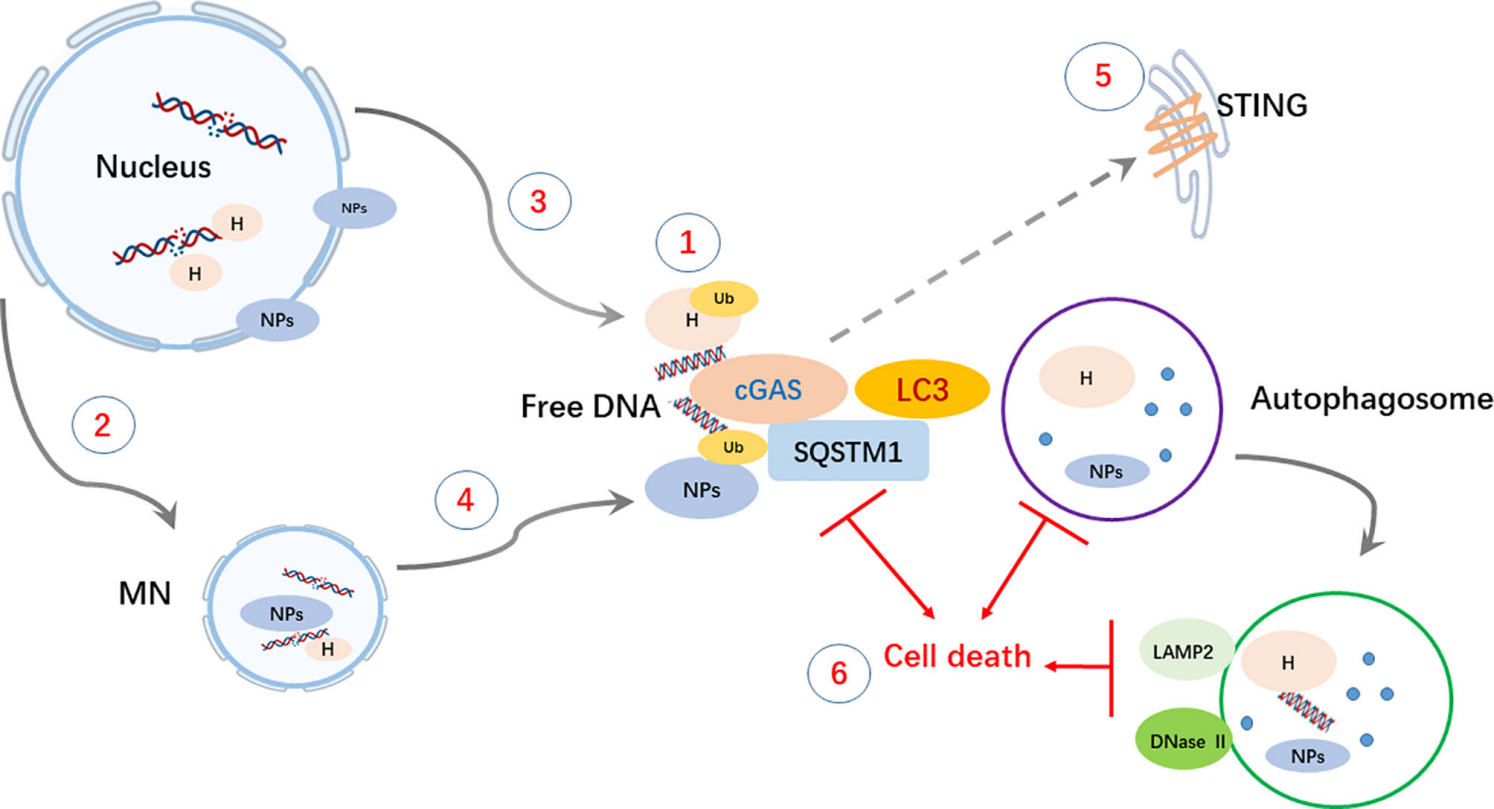
Schematic summary of the main findings. Cytoplasmic DNA autophagy is initiated by the binding of cGAS to free DNA and may be coordinately accompanied by the recognition of ubiquitinated Ub) histones H) or nuclear proteins NPs) by SQSTM1. ① Cytoplasmic DNA and proteins) could be derived from either DNA replication-related nuclear damage ② and consequent MN formation ③, which would undergo collapse owing to its unscheduled replication ④. ⑤ The degradation of STING could be involved in autophagy and result in a failure to activate SASP activity, but the role of STING in DNA autophagy still needs further exploration. ⑥ Generally, in autophagy, free DNA and proteins are enclosed in autophagosomes *via* LC3 lipidation and degraded after fusion with LAMP2- and DNase II-containing lysosomes. The clearance of cytoplasmic DNA by autophagy could play a protective role in maintaining the growth and survival of cancer cells, and thus, disruption of the process could lead to caspase-independent cell death CICD).

In summary (**Figure 6**), our investigation revealed DNA autophagy in breast cancer cells with high MN formation. Autophagy of genomic DNA in the cytosol could be mediated by cGAS but is usually coordinated with other autophagic mediators. Cytoplasmic DNA could be derived from DNA replication-induced damage and MN collapse. The clearance of cytoplasmic DNA could be necessary for cancer cell growth and survival. Thus, autophagic inhibition could be a potential therapeutic approach for cancer cells with high DNA autophagic activity. Nevertheless, we acknowledged that our major evidences came from BT-549, there will be further work in BT-549 like breast or other cancer cells to solid our conclusions.

## Data Availability Statement

The original contributions presented in the study are included in the article/**Supplementary Material**. Further inquiries can be directed to the corresponding author.

## Author Contributions

MY and YW performed experiments and analyzed the data. YNC, NM, and YJC conducted the statistical analyses. BZ and MY designed the study and wrote the manuscript. YW and HL revised manuscript. HZ, SZ, LN, and LL assisted some experiments. All authors contributed to the article and approved the submitted version.

## Funding

This project was supported by the National Natural Science Foundation of China No. 81872018) and the Key Project from the Chinese Ministry of Science and Technology No. 2017 FC0110200).

## Conflict of Interest

The authors declare that the research was conducted in the absence of any commercial or financial relationships that could be construed as a potential conflict of interest.
