# Supplementary material for: Autophagy-Mediated Clearance of Free Genomic DNA in the Cytoplasm Protects the Growth and Survival of Cancer Cells
Source: Front Oncol. 2021 May 26;11:667920. doi: 10.3389/fonc.2021.667920 (PMC8189927; doi:10.3389/fonc.2021.667920)
Supplement: Supplementary file 1 [file DataSheet_1.docx]

| Antibody | Source | IF Dilution ratio | WB Dilution ratio |
| --- | --- | --- | --- |
| STAT6 | Santa Cruz Biotechnology (sc-271770) | 1/1500 | 1/500 |
| cGAS | Santa Cruz Biotechnology (sc-515777) | 1/300 | 1/500 |
| translocated promoter region, nuclear basket protein | Santa Cruz Biotechnology (sc-101294) | 1/300 |  |
| Lamin B1 | Santa Cruz Biotechnology (sc-374015) | 1/300 |  |
| Lamin B1 | Abcam (ab16048) | 1/1000 |  |
| STING | Abcam (ab181125) | 1/1000 | 1/1000 |
| Histone core | Abcam (ab7832) |  | 1/1000 |
| DNase II | Abcam (ab8119) | 1/500 | 1/1000 |
| Brdu | Abcam (#152095) | 1/1000 |  |
| Nup153 | Abcam (ab24700) | 1/500 |  |
| Mab414 | Ab24609 | 1/500 | 1/1000 |
| phospho-histone γH2AX (Ser139) | Millipore (#JBW301) | 1/1000 | 1/1000 |
| RPA2 | Millipore (MABE285) | 1/500 | 1/1000 |
| LAMP2 | Bioworld (BS6867) | 1/500 | 1/1000 |
| IRF3 | Bioworld (BS6921) | 1/500 | 1/500 |
| Anti-STING (phospho Ser365) | Cell Signaling Technology (#D8F4W) |  | 1/1000 |
| Anti-IRF3 (phospho Ser396) | Cell Signaling Technology (#4D4G) |  | 1/1000 |
| Anti-ATM (phospho S1981) | Bioworld (BS94001) | 1/100 | 1/500 |
| Anti-ATR (phospho T1989) | Abcam (ab227851) | 1/500 | 1/1000 |
| LC3 | MBL (PM036) | 1/500 | 1/1000 |
| SQSTM1 | MBL (PM045) | 1/500 | 1/1000 |
| MCM7 | Proteintech (1125-1-AP) | 1/500 |  |
| LaminA/C | Bioworld (BS1446) | 1/200 | 1/500 |
| Anti-LaminB Receptor (LBR) | Millipore (MABT831) |  | 1/1000 |
| β-actin | Origene (TA-09) |  | 1/1000 |
| Flag-tag | Origene (TA-05) | 1/500 | 1/1000 |
| Flag-tag | Bioworld(AP0007) | 1/200 | 1/1000 |

Supplementary Table1：The Antibody information

*All antibodies are diluted with TBST (Tris-Buffered Saline and Tween 20)_­­_
