# Supplementary material for: Autophagy-Mediated Clearance of Free Genomic DNA in the Cytoplasm Protects the Growth and Survival of Cancer Cells
Source: Front Oncol. 2021 May 26;11:667920. doi: 10.3389/fonc.2021.667920 (PMC8189927; doi:10.3389/fonc.2021.667920)
Supplement: Supplementary file 2 [file DataSheet_2.docx]

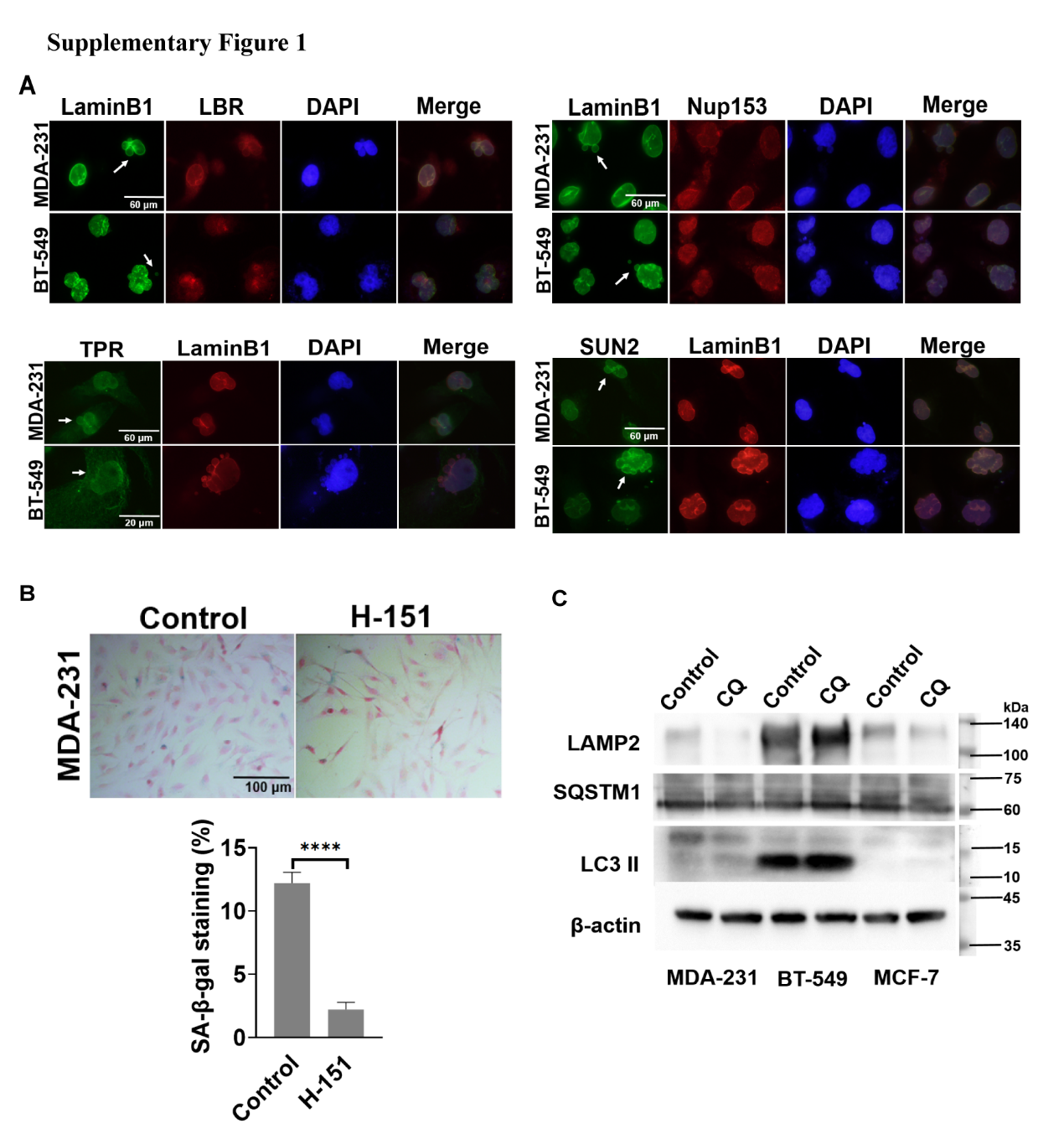


**Supplementary FIGURE 1** | **(A)** The analysis of composition of MN nuclear membrane in breast cancer cells. MDA-231 and BT-549 cells were subjected to immunofluorescent staining for LaminB1 and LBR, TPR, Nup153, or Sun2, respectively. Arrows indicate MN. **(B)** Inhibitor of STING reduced senescent phenotypes of MDA-231 cells. Left panels: MDA-231 cells were treated with H-151 (2 μmol/L) for 24 hours and stained by SA-β-gal. Right panel: SA-β-gal positive cells were counted and calculated as percentage of total cells. The level of statistical significance was < 0.0001 (****). The experiments were independently repeated five times, and the images are representatives of repeated experiments. Data are presented as mean ± SEM. **(C)** The effects of autophagic inhibition on the expression of autophagy-related proteins in breast cancer cells. MDA-231, BT-549 and MCF-7 cells were treated with CQ (50 μmol/L) for 4 hours, respectively, and LAMP2, SQSTM1, LC3 II was measured by Western blotting, respectively. The experiments were independently repeated three times, and the images are representatives of repeated experiments. Data are presented as mean ± SD.


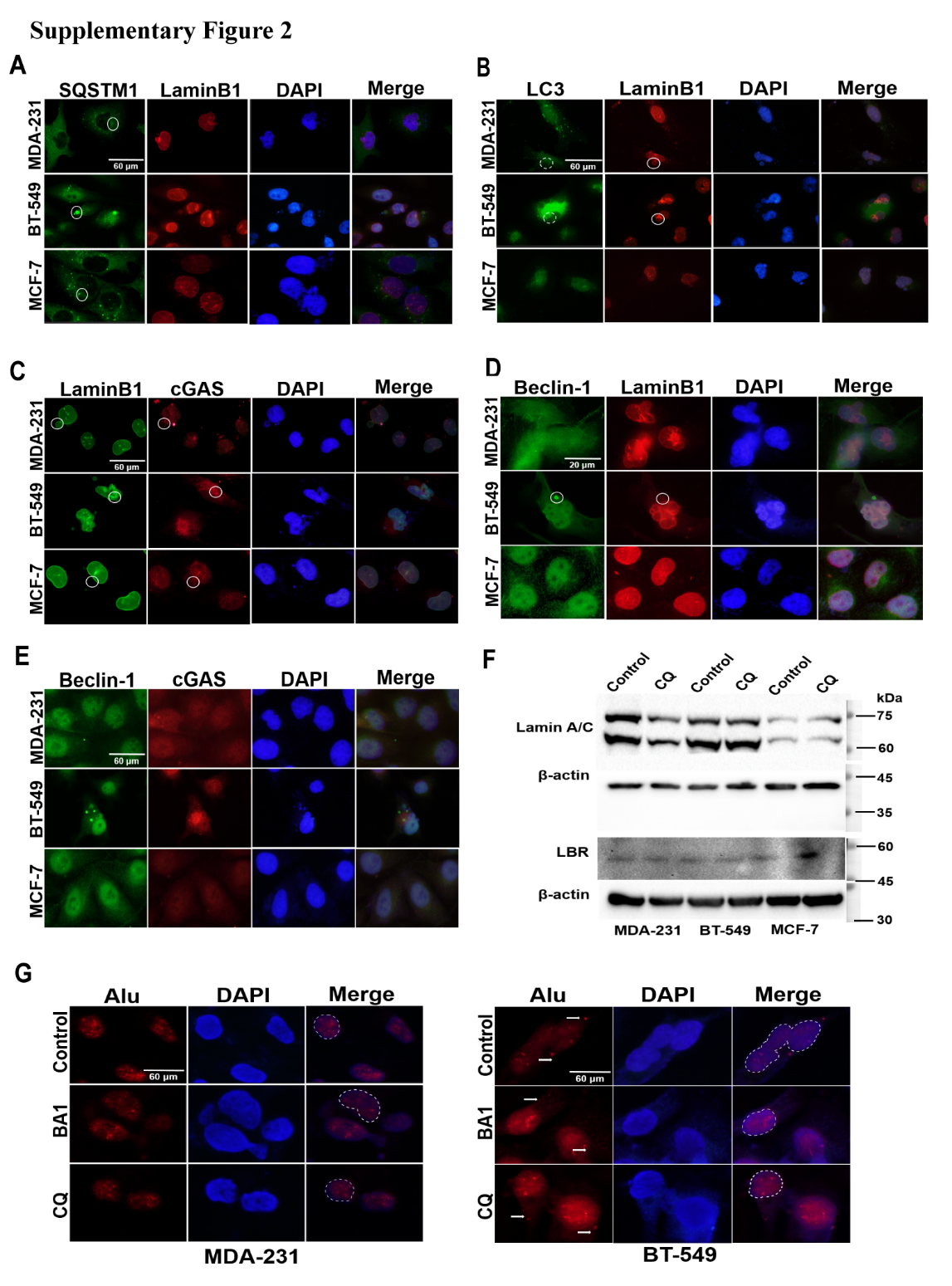


**Supplementary FIGURE 2** | The expression and distribution of cGAS, SQSTM1, LC3 and Beclin-1 in MN of MDA-231, BT-549 and MCF-7 cells. **(A)** MDA-231, BT-549 and MCF-7cells cells were subjected to double immunofluorescent staining of SQSTM1 (green) and LaminB1 (red). MN with co-localization was labeled by circles. **(B)**The detection of LC3 (green) and LaminB1 (red) in MN of MDA-231 and BT-549 and MCF-7 cells. MN with co-localization was labeled by solid circles. **(C)** The expression and distribution of LaminB1 (green) and cGAS (red) in MDA-231, BT-549 and MCF-7cells. MN with co-localization was labeled by circles. **(D)** Beclin-1(green) and LaminB1 (red) were doubly stained in MDA-231, BT-549 and MCF-7cells. MN with co-localization was labeled by circles. **(E)** MDA-231, BT-549 and MCF-7 cells were double stained in immunofluorescent by Beclin-1 and cGAS. **(F)** The effects of autophagic inhibition on the expression of nuclear laminar proteins in breast cancer cells. MDA-231, BT-549 and MCF-7 cells were treated with CQ (50 μmol/L) for 4 hours, respectively, and LaminA/C or LBR was measured by Western blotting, respectively. (**G**) Alu-based FISH was performed in MDA-231 and BT-549 cells with or without the presence of CQ (50 μmol/L) or bafilomycinA1 (10 nmol/L) for 36 hours, respectively. Arrows indicate cytoplasmic DNA. All the experiments were repeated at least three times independently and the images are representatives of repeated experiments.


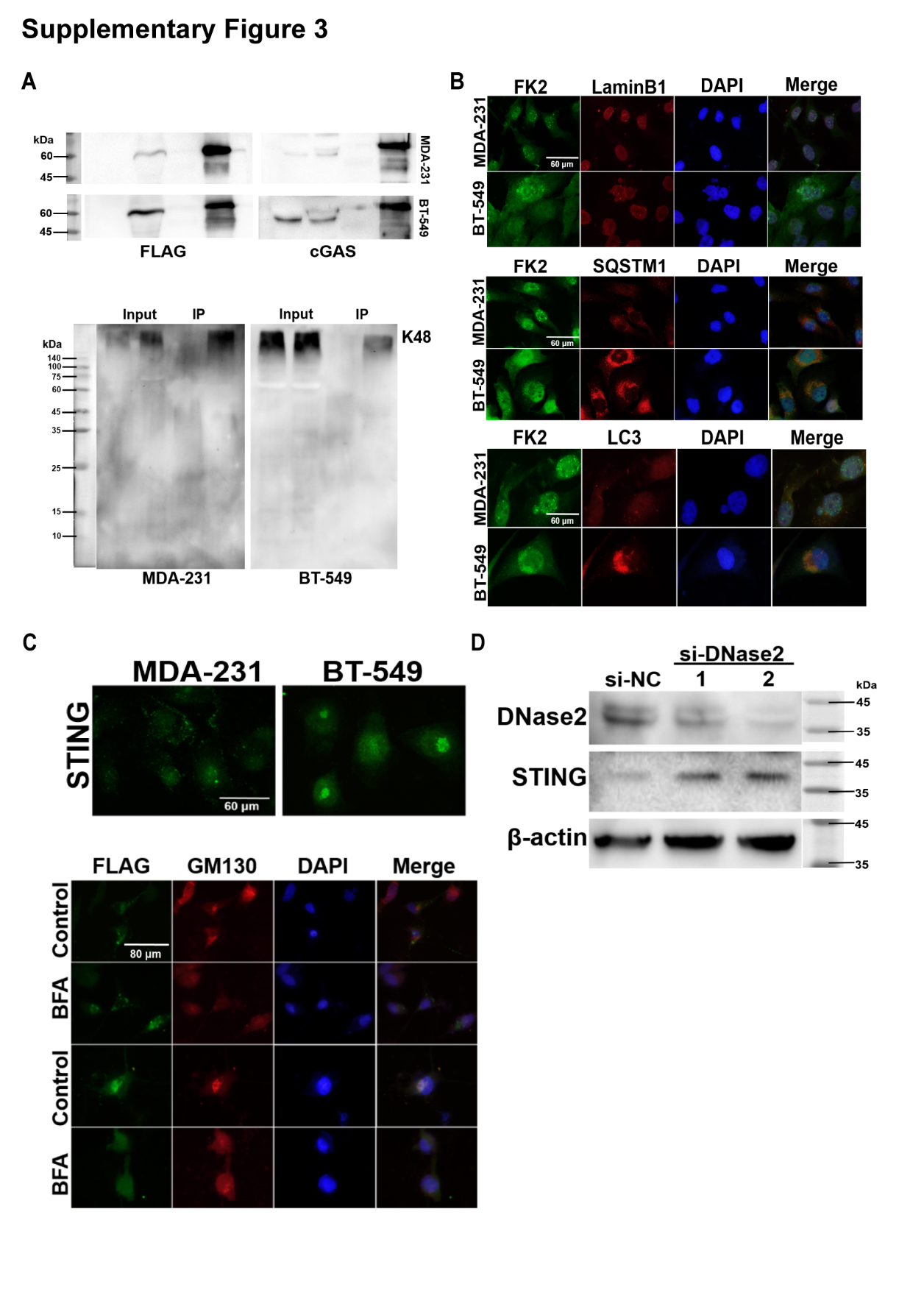


**Supplementary FIGURE 3** | **(A)** The analysis on the potential complex of SQSTM1 and cGAS in breast cancer cells. MDA-231 and BT-549 cells were transfected with Flag-SQSTM1 (or vector) for 36 hours, respectively, and then cytoplasmic extracts were isolated and immune-precipitated by Flag beads as described in Materials and Methods. Upper panel: Flag and cGAS were detected by Western blotting, respectively. Lower panel: Anti-K48 ubiquitin was detected by Western blotting, respectively. **(B)**The analysis on ubiquitinated proteins in breast cancer cells. The double staining of FK2 (green) with LaminB1 (red), LC3 (red) and SQSTM1 (red), respectively. **(C)** The distribution of STING in breast cancer cells. Upper panel: MDA-231 and BT-549 cells were stained by STING. Lower panel: the cells were transfected with Flag-STING for 24 hours, and treated with the BFA (0.66 μmol/L) for 4 hours, then were double stained with Flag (green) and GM130 (red). **(D)** Silencing of *DNase II* increased the level of STING. BT-549 cells were transfected si-DNase II (1, 2), respectively, and DNase II and STING were measured by Western Blotting. All the experiments were independently repeated three times, and the images are representatives of the repeated experiments.


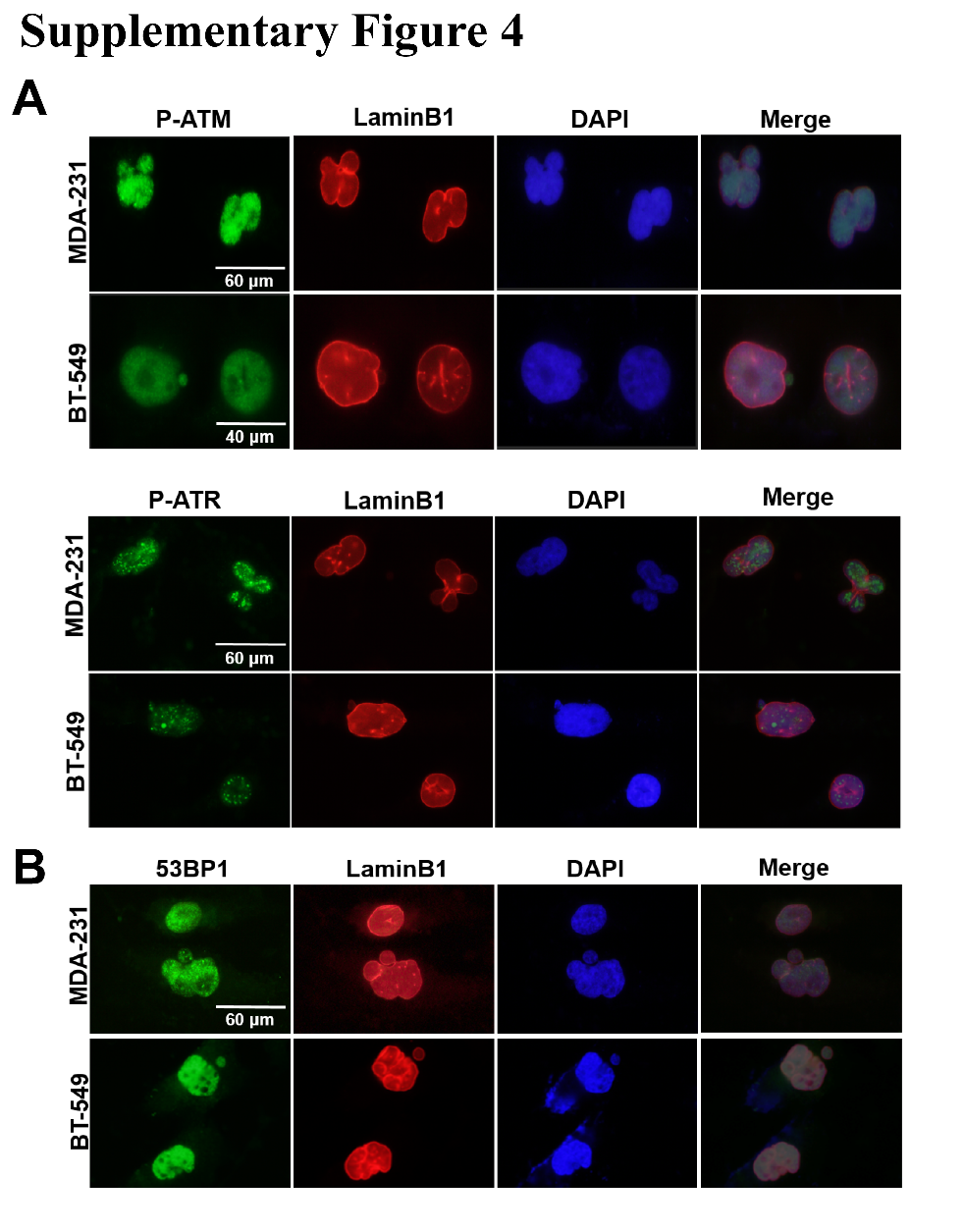


**Supplementary FIGURE 4** |The analysis on DNA damage in MN in breast cancer cells. **(A)** The double staining of 53BP-1 and LaminB1 was performed in MDA-231 and BT-549 cells, respectively. **(B)** Double staining of 53BP1 with and Lamin B1 was performed in MDA-231 and BT-549 cells, respectively. The positive staining in nucleui and MN were evaluated, respectively. All the experiments were independently repeated three times, The images are representatives of repeated experiments.


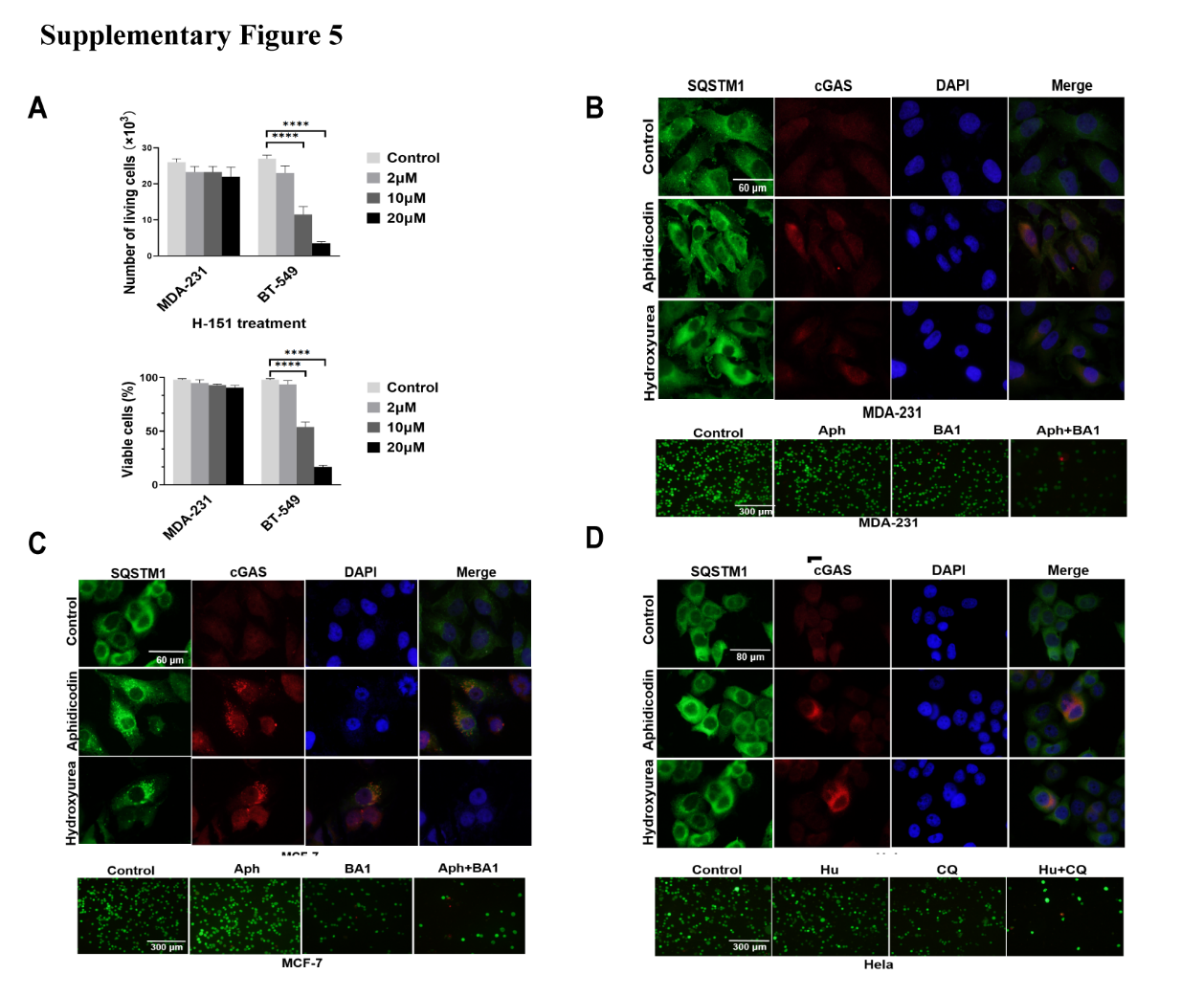


**Supplementary FIGURE 5** | **(A)** The antagonist of STING in the autophagy. MDA-231 and BT-549 cells were treated with H-151. an antagonist of STING in indicated concentrations for 48 hours and cell viability was measured by Trypan blue exclusion assay. The experiments were repeated three times independently. The level of statistical significance was < 0.0001 (****). Data are presented as mean ± SEM. **(B-D),** Enhancement of DNA damage sensitized cancer cells to autophagy inhibitors. MCF-7, MDA-231, HeLa cells treated with Hydroxyurea (0.5 mmol/L) or Aphidicolin (1μmol/L) for 4 hours and then were further incubated in the presence of CQ (50 μmol/L) or bafilomycinA1 (10 nmol/L) for 36 hours. The cell viability was analyzed by Live and dead assay (lower panels). The expression and distribution induced by Hydroxyurea (0.5 mmol/L) or Aphidicolin (1μmol/L) in MCF-7, MDA-231, HeLa cells were analyzed by double staining of SQSTM1 and cGAS (upper panels), respectively. Each of experiment was done in triplicate and all experiments were independely repeated at least three times. The images are representatives of repeated experiments.


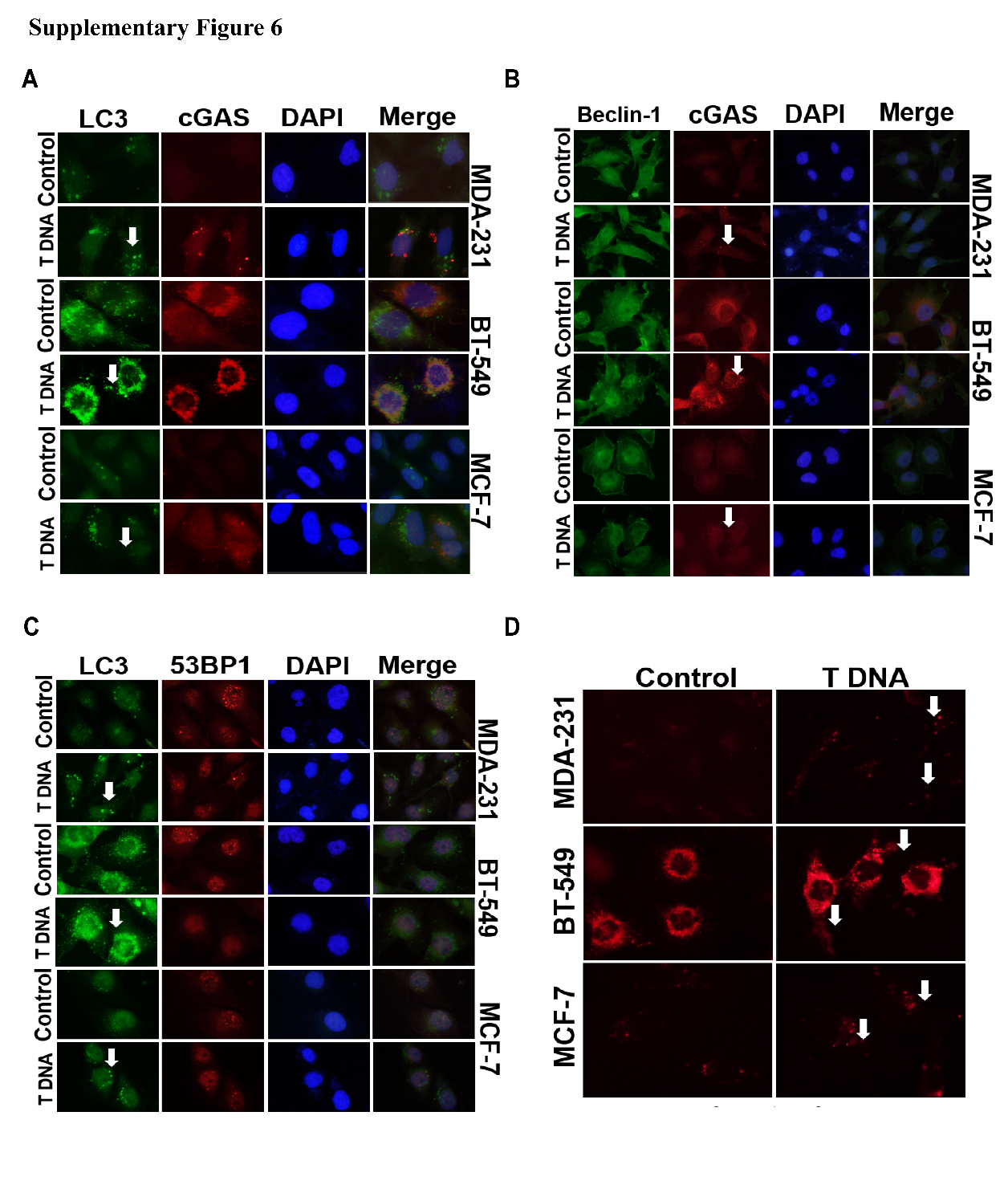


**Supplementary FIGURE 6** |The analysis on exogenous DNA and autophagy in breast cancer cells. MDA-231, BT-549 cells or MCF-7 cells were transfected with the genomic DNA (1μg/ml) from HeLa cells 36 hours, respectively, and subsequent staining was performed. **(A)** The double staining of LC3 and cGAS. Arrows indicate increased cytoplasmic vesicles. **(B)** The double staining of Beclin-1 and cGAS, and arrows indicate increased cytoplasmic vesicles. **(C)**The double staining of LC3 and 53BP1, and arrows indicate increased cytoplasmic vesicles. (**D)** Lysosomes were labeled by LysoTracker. Arrows indicate increased cytoplasmic vesicles. All the experiments were independently repeated three times.The images are representatives of repeated experiments.
